# Identification of ColR binding consensus and prediction of regulon of ColRS two-component system

**DOI:** 10.1186/1471-2199-10-46

**Published:** 2009-05-16

**Authors:** Paula A Kivistik, Rait Kivi, Maia Kivisaar, Rita Hõrak

**Affiliations:** 1 Estonian Biocentre and Institute of Molecular and Cell Biology, Tartu University, 51010 Tartu, Estonia

## Abstract

**Background:**

Conserved two-component system ColRS of *Pseudomonas *genus has been implicated in several unrelated phenotypes. For instance, deficiency of *P. putida *ColRS system results in lowered phenol tolerance, hindrance of transposition of Tn*4652 *and lysis of a subpopulation of glucose-grown bacteria. In order to discover molecular mechanisms behind these phenotypes, we focused here on identification of downstream components of ColRS signal transduction pathway.

**Results:**

First, highly similar ColR binding sites were mapped upstream of outer membrane protein-encoding *oprQ *and a putative methyltransferase-encoding PP0903. These two ColR binding sequences were used as an input in computational genome-wide screening for new potential ColR recognition boxes upstream of different genes in *P. putida*. Biological relevance of a set of *in silico *predicted ColR-binding sites was analysed *in vivo *by studying the effect of ColR on transcription from promoters carrying these sites. This analysis disclosed seven novel genes of which six were positively and one negatively regulated by ColR. Interestingly, all promoters tested responded more significantly to the over-expression than to the absence of ColR suggesting that either ColR is limiting or ColS-activating signal is low under the conditions applied. The binding sites of ColR in the promoters analysed were validated by gel mobility shift and/or DNase I footprinting assays. ColR binding consensus was defined according to seven ColR binding motifs mapped by DNase I protection assay and this consensus was used to predict minimal regulon of ColRS system.

**Conclusion:**

Combined usage of experimental and computational approach enabled us to define the binding consensus for response regulator ColR and to discover several new ColR-regulated genes. For instance, genes of outer membrane lipid A 3-O-deacylase PagL and cytoplasmic membrane diacylglycerol kinase DgkA are the members of ColR regulon. Furthermore, over 40 genes were predicted to be putatively controlled by ColRS two-component system in *P. putida*. It is notable that many of ColR-regulated genes encode membrane-related products thus confirming the previously proposed role of ColRS system in regulation of membrane functionality.

## Background

Two-component signal systems are the main means for sensing the changing environment in a prokaryotic world [1]. Typically, bacterial signal transduction systems consist of two components, a sensor histidine kinase and a response regulator. A specific compound or a physicochemical property of the environment acts as a signal triggering the activation of a membrane embedded sensor, which in turn autophosphorylates and thereafter passes the signal to a response regulator via phosphoryl group transfer [2]. Phosphorylated response proteins mostly act as DNA binding transcription factors by activating or repressing the expression of target genes.

The number of genes for two-component proteins varies greatly between the genomes of sequenced bacteria, being for instance zero in case of *Mycoplasma genitalium *and 62 in a well-known model organism *Escherichia coli *[3]. The abundance of two-component systems seems to correlate with environmental and pathogenic versatility of a bacterium. *Pseudomonas *bacteria that colonise different habitats such as soil, water, plants and animal tissues, possess many two-component signal systems to cope with various environments. For example, over a hundreds genes encoding two-component system proteins are present in the genome of *Pseudomonas aeruginosa *[4].

The ColRS two-component signal transduction system, intrinsic to *Pseudomonas *species, consists of a sensor kinase ColS and a response regulator ColR. *colRS *operon is well-conserved among the sequenced members of *Pseudomonas *genus [5] suggesting that ColRS system could be important to these bacteria. ColRS pathway was first characterised in *P. fluorescens *as a system involved in the ability of bacteria to competitively colonise plant roots [6]. Our group observed a totally different function for ColRS system as transposition of Tn*4652 *was inhibited in phenol-starving *colR*- and *colS*-deficient *P. putida *[7]. Additionally, recently, we demonstrated that *colR*-deficient *P. putida *is sensitive to phenol [8] and displays a serious defect on solid glucose medium where a subpopulation of the mutant lyses [9]. ColRS system was also shown to be important in resistance of *P. putida *to divalent metal ions, especially Mn^2+ ^[10]. Importantly, the precise checkpoint of a response regulator ColR remained unclear in case of all these ColR-dependent phenotypes. However, two recent publications suggest that seemingly unrelated phenotypes of *colR*-deficient *P. fluorescens *and *P. putida *can most probably be explained by the compromised cell membranes of *colR *mutants. Namely, the first ColR-regulated genes that were identified in these two species, encoded different membrane functions. We demonstrated that *oprQ *and *algD*, which encode a porin protein and an exopolysaccharide alginate biosynthesis enzyme, respectively, are under the direct control of ColR in *P. putida *[8]. Concomitantly, de Weert *et al *[11] reported for *P. fluorescens *that an operon downstream of *colRS *hypothetically coding for membrane associated proteins, methyltransferase (*orf222*) and lipopolysaccharide kinase (*inaA/wapQ*), is also regulated by ColR.

In spite of several recent studies disclosing the ColRS system as an important signal transduction pathway for pseudomonads, little is known about the downstream components of this signal system. Only two target genes of ColR have been identified in *P. putida *[8] and our unpublished data show that neither *oprQ *nor *algD *are involved in phenotypes characteristic to *colR *mutant. Thus, more ColR-regulated genes should be present in the genome of *P. putida*. In this study we aimed to determine the ColR binding consensus sequence and to use it in the screen of *P. putida *genome for the presence of new potential ColR binding sites, which would predict new ColR target genes and operons.

## Results

### ColR (PP0901) regulates the expression of PP0903

**Figure 1:**
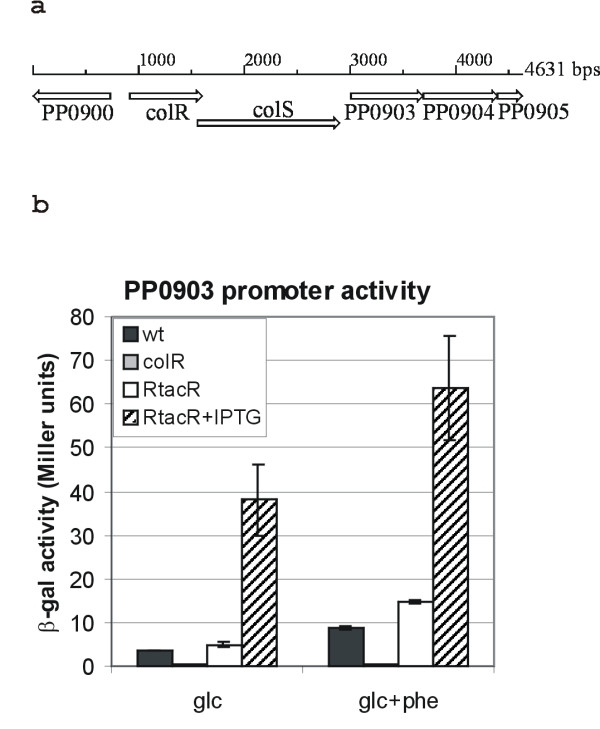
**ColR activates the expression of its downstream-locating PP0903**. (a) Organization of genes in *P. putida colRS *locus. (b) β-galactosidase (β-gal) activities measured in *P. putida *wild-type strain PaW85 (wt), *colR*-defective strain PaWcolR (colR) and *colR*-defective strain PaWRtaccolR complemented with *colR *gene under the control of the inducible P_tac _promoter (RtacR). All strains carried the reporter plasmid with the promoter of PP0903. ColR expression in strain PaWRtaccolR was induced with 0.5 mM IPTG (RtacR+IPTG). Bacteria were grown either on glucose (glc) or glucose plus 2.5 mM phenol (glc+phe) minimal plates. Data (means and standard deviations) from at least three independent experiments are presented. For promoter probe vector p9TT_B_lacZ, the basal level of β-galactosidase activity was less than 0.5 Miller units.

The expression of genes downstream of *colRS *operon is regulated by ColR in *P. fluorescens *[11]. This region of genome is well conserved among *Pseudomonas *bacteria [5] and therefore we presumed that the operon downstream of *colRS *(Fig. 1a) could be regulated by ColRS system in *P. putida *as well. This downstream operon consists of three genes (Fig. 1a). The first two, PP0903 coding for a putative methyltransferase and PP0904 coding for a lipopolysaccharide kinase InaA, are conserved among all 15 fully sequenced pseudomonads [5]. Putative orthologs of the third open reading frame PP0905 encoding for a hypothetical protein are present downstream of *inaA *in 13 sequenced members of *Pseudomonas *genus. In order to test whether these genes belong to ColR regulon in *P. putida*, we cloned the upstream region of PP0903 in front of *lacZ *gene in a promoter probe plasmid p9TT_B_lacZ and compared plasmid-encoded β-galactosidase activities in wild-type *P. putida *strain PaW85 and in its ColR-deficient derivative. Our unpublished results show that ColR-dependent phenotypes are more pronounced on solid medium if compared to that in liquid medium. Therefore, all enzyme activities presented in this study were measured from solid-medium-grown bacteria. The obtained data showed that ColR is a positive effector of PP0903 expression since β-galactosidase activity was comparable to the basal level of promoter probe vector p9TT_B_lacZ in a *colR*-deficient strain but was 10-fold higher in a wild-type strain (Fig. 1b). As our previous study showed that ColR-regulated *oprQ *and *algD *promoters were affected by phenol [8], the β-galactosidase activity was also measured in the presence of phenol. Again, the activity of PP0903 promoter remained indistinguishable from the basal level of vector in the *colR*-mutant strain (Fig. 1b). However, phenol significantly enhanced the activity of PP0903 promoter in a wild-type strain where ColR is present (Fig. 1b). To confirm the role of ColR in the activation of PP0903 promoter, β-galactosidase measurements were also performed in *colR*-mutant derivative PaWRtaccolR where the expression of ColR was inducible from P_*tac *_promoter with addition of IPTG. The activity of PP0903 promoter was already recovered in PaWRtaccolR strain without the induction of *colR *(Fig. 1b). This was expected as immunoblot analysis of ColR expression had previously revealed significant leakiness of P_*tac *_promoter in that particular strain [7]. Over-expression of ColR resulted in a strong enhancement of PP0903 promoter over the wild-type level both on glucose and glucose plus phenol medium (Fig. 1b). Hence, response regulator ColR is indeed necessary for up-regulation of PP0903 promoter in *P. putida*. Strong induction of the promoter by ColR over-expression suggests that either the amount of ColR is limiting in the wild-type cells or the ColS-activating signal is low in tested conditions. Data also imply that the impact of ColR on the PP0903 activation becomes more prominent in bacteria experiencing phenol-caused stress.

### ColR binding sites in the promoter regions of *oprQ *(PP0268) and PP0903

**Figure 2:**
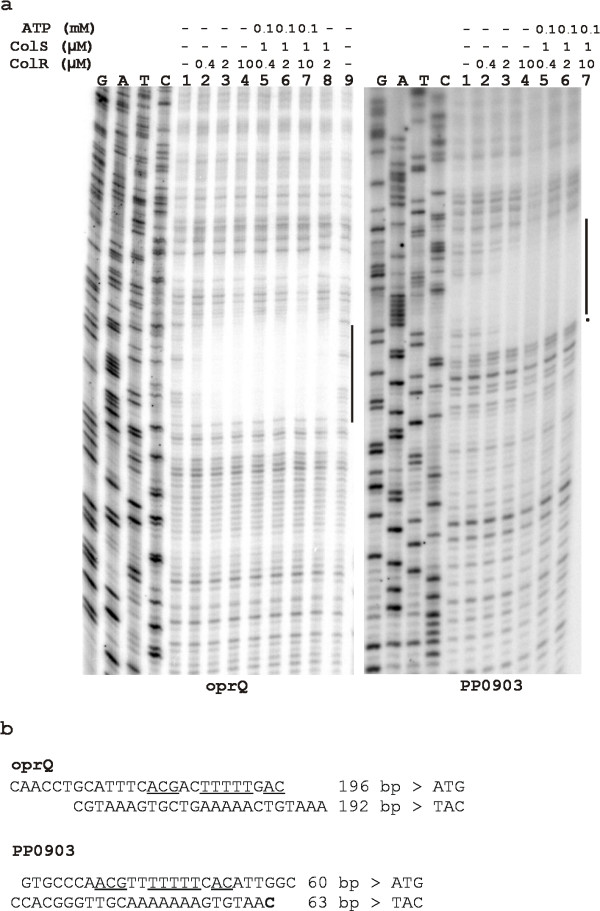
**ColR binding sites in the promoter regions of *oprQ *and PP0903**. (a) DNase I footprinting assay performed on *oprQ *and PP0903 promoter DNA. Dideoxy-chain termination sequences corresponding to the probes are shown in the order of G, A, T and C. Concentrations of DNA-binding protein ColR, its cognate kinase ColS, and phosphoryl-donor ATP are shown above each lane. Vertical black lines indicate ColR-protected regions and dot represents a site hypersensitive to DNase I cleavage. (b) Sequences of ColR-protected regions in the promoters of *oprQ *and PP0903. A site hypersensitive to DNase I cleavage in the presence of ColR is in bold. Nucleotides identical in two ColR binding sites are underlined. Distance of the ColR-binding site from the start codon is presented.

One possible way to find new target genes of a transcriptional regulator is to computationally analyze the whole genome of an organism. As a certain binding consensus of a transcription factor is needed for that, we aimed to specify the exact binding sites of ColR in the promoters under its control. Our previous paper demonstrated that purified histidine-tagged ColR bound *in vitro *to the promoters of *oprQ *and *algD *in a gel mobility shift assay [8]. Similar analysis with PP0903 promoter showed that ColR also directly interacts with this promoter DNA. In order to map the ColR binding sites we performed a DNase I footprinting assay to the DNAs of *oprQ*, *algD *and PP0903 promoter fragments labelled with P^32^. Fig. 2a illustrates the ColR-recognized regions in the promoters of *oprQ *and PP0903. ColR protected a 24-to-26-bp-long area in both promoters (Fig. 2b) depending on a strand analysed. ColR binding sites reside about 190 and 60 nucleotides upstream of translation start codons of *oprQ *and PP0903, respectively. The binding of ColR to PP0903 promoter resulted in the appearance of a hypersensitive site for DNase I just next to the ColR-protected region (Fig. 2a). Data also showed that the phosphorylated form of ColR (ColR~P) binds to the DNA with higher affinity than the unphosphorylated ColR. However, the effect of phosphorylation was stronger in case of PP0903 compared to *oprQ *(Fig. 2a).

Despite the fact that ColR bound to the promoter DNA of *algD *in a gel mobility shift assay [8] we could not detect a ColR-binding site in this DNA by DNase I footprinting (data not shown). This suggests that the binding dynamics of response regulator ColR could be somewhat different if *algD *promoter is compared to the promoters of *oprQ *and PP0903. Most probably ColR recognition sequences in the *algD *promoter diverge from ColR consensus (see Discussion).

### Computer search for potential ColR binding sites revealed several new ColR-regulated genes

**Figure 3:**
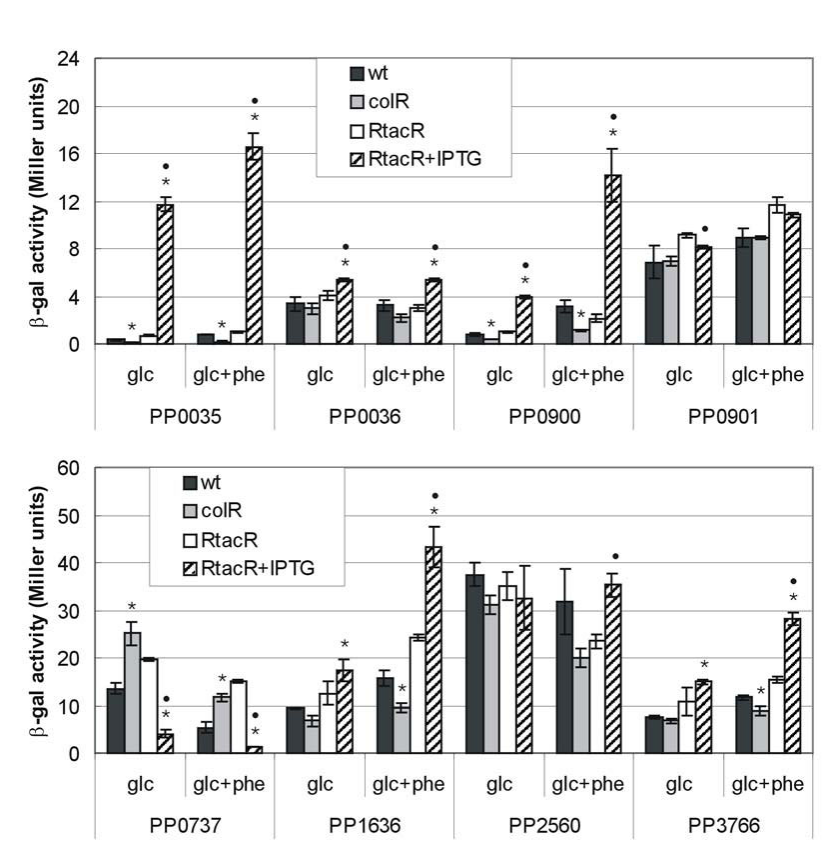
**Effects of ColR on the expression of a set of computationally predicted promoters**. β-galactosidase (β-gal) activities measured in wild-type (wt), *colR*-defective (colR) and *colR*-defective strain PaWRtaccolR complemented with *colR *gene under the control of the P_tac _promoter (RtacR). *P. putida *strains carried reporter plasmids with promoter regions of following genes: PP0035, PP0036, PP0900, PP0901, PP0737, PP1636, PP2560 or PP3766. Bacteria were grown either on glucose (glc) or glucose plus 2.5 mM phenol (glc+phe) minimal plates. ColR expression in strain PaWRtaccolR was induced with 0.5 mM IPTG (RtacR+IPTG). Data (means and standard deviations) from at least three independent experiments are presented. For promoter probe vector p9TT_B_lacZ, the basal level of β-galactosidase activity was less than 0.5 Miller units. Asterisks above the bars indicate statistically significant differences (*p *< 0.05 according to t-test) between the promoter activities of particular strain and wild-type. Dots indicate significant differences between PaWRtaccolR strain and PaWRtaccolR strain grown with IPTG.

Comparison of ColR recognition sequences in the promoters of *oprQ *and PP0903 (Fig. 2b) shows clearly that these two sequences are similar. The most obvious common feature is a central T-track, 5-bp and 7-bp long in case of *oprQ *and PP0903, respectively (Fig. 2b). In total 10 identical nucleotides are present in these two ColR binding sites allowing to deduce a 13-bp-long consensus motif for ColR binding – ACGNNTTTTTNAC. High similarity of the two ColR binding boxes suggested that a genome-wide computer search could reveal new potential target genes of ColR. Thus, sequences similar to identified ColR binding sites were searched from the genome of *P. putida *KT2440 using two free-access web-servers, PredictRegulon [12,13] and Virtual Footprint [14,15]. Several inquiries with varying input sequence length and strand orientation were performed. Results of both programmes were compared and identical hits were chosen for experimental validation. Actually, the prediction and identification of new ColR binding sites presented below was a step-by-step process meaning that new predictions were made with every additional confirmed ColR binding site (see Methods). For experimental evaluation of the computer-predicted putative ColR-regulated promoters, they were cloned into a promoter probe plasmid p9TT_B_lacZ upstream of the *lacZ *gene. If a predicted ColR-binding site was located between two divergently transcribed genes, then this DNA region was cloned upstream of the reporter in both orientations (promoters of PP0035/0036, PP0900/0901 and PP2560/2561). β-galactosidase activities were measured in wild-type and ColR-deficient *P. putida *cells grown overnight on glucose and glucose plus phenol solid media. Additionally, since promoter activity of PP0903 was strongly enhanced by over-expression of ColR (Fig. 1b) all promoters were tested also in a *P. putida *strain PaWRtaccolR where the expression of ColR can be induced from P_tac _promoter. Results of β-galactosidase assay show that promoters of PP0035, PP0036, PP0737, PP0900, PP1636, PP2560 and PP3766 are responsive to the absence and/or to the over-expression of ColR (Fig. 3). All promoters except PP0737 are positively affected by ColR. If promoters were tested in the presence of 2.5 mM phenol then the effects of ColR-deficiency and over-expression became more evident in case of most promoters. For instance, while promoters of PP0036, PP0900, PP1636, PP2560 and PP3766 were only barely affected by the absence of ColR in the glucose-grown cells, then the impact of ColR upon these promoters became obvious in the glucose plus phenol-grown bacteria. It is also remarkable that all promoters analysed responded significantly more to the over-expression than to the absence of ColR. Namely, ColR over-expression clearly enhanced the activities of promoters that are under its positive control (Fig. 3). Especially drastic activation was seen in case of PP0035 where over-expressed ColR caused 30-fold increase in the promoter activity when compared to that in a wild-type strain (Fig 3). At the same time, the activity of PP0035 promoter was only 3-fold lower in glucose-grown *colR *mutant than in wild-type. Analogous to that, the over-expression of ColR lowered the activity of negatively ColR-affected PP0737 promoter about 3-fold but the lack of ColR affected this particular promoter less than 2-fold (Fig. 3). This data strongly suggest that either ColR amount is limiting in wild-type *P. putida *to affect significantly the expression of analysed target promoters or the signal perceived and transduced by ColS is low under the conditions employed by us.

It is important to point out that one new ColR-regulated gene, PP0900, locates just upstream of *colRS *genes and is transcribed divergently from PP0901 encoding *colR *(Fig. 1a). Thus, the putative ColR-binding site affecting the expression of PP0900 is actually located between PP0900 and *colR*. Analysis of this particular promoter in the direction of *colR *(PP0901) did not reveal clear ColR-responsiveness under any conditions examined (Fig. 3). Therefore we conclude that ColR does not auto-regulate its own expression.

### ColR binding sites in the promoters of predicted target genes

**Figure 4:**
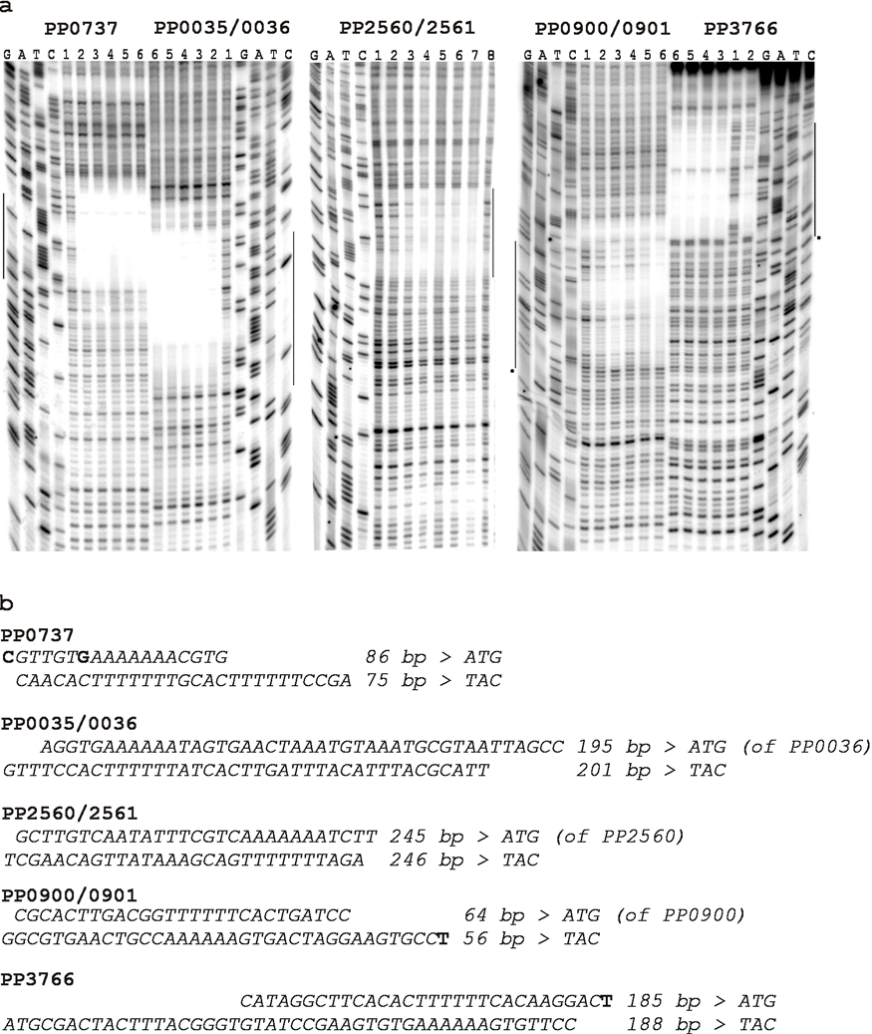
**ColR-protected regions in the promoters of PP0737, PP0035/0036, PP2560/2561, PP0900/0901 and PP3766**. (a) DNase I footprinting assay performed on promoter DNAs of PP0737, PP0035/0036, PP2560/2561, PP0900/0901 and PP3766. Lane 1 is always a DNase I-only ladder, following lanes represent different concentrations of DNA-binding protein ColR in a non-phosphorylated (lanes 2 and 3; in case of PP2560/2561 lanes 2 to 4) and phosphorylated state (lanes 4 to 6; in case of PP2560/2561 lanes 5 to 7). Concentrations of ColR in reactions with ColS and ATP were 0.6 μM, 3 μM and 15 μM if DNA upstream of PP3766, PP0900/0901 or PP2560/2561 was used as a binding target, but 0.5 μM, 2.5 μM and 12.5 μM in case of PP0737 and PP0035/0036 promoter DNA. Concentration of ColR-phosphorylating ColS was always 1 μM and ATP was 0.1 mM. Only the two higher concentrations of ColR are used in the binding reactions with non-phosphorylated ColR (lanes 2 and 3), except for PP2560/2561 where all three ColR concentrations are presented (lanes 2 to 4). (b) Sequences of the ColR-protected regions in the promoters of PP0737, PP0035/0036, PP2560/2561, PP0900/0901 and PP3766. Distance from the start codon is shown and the sites hypersensitive to DNase I cleavage in the presence of ColR are highlighted in bold.

**Figure 5:**
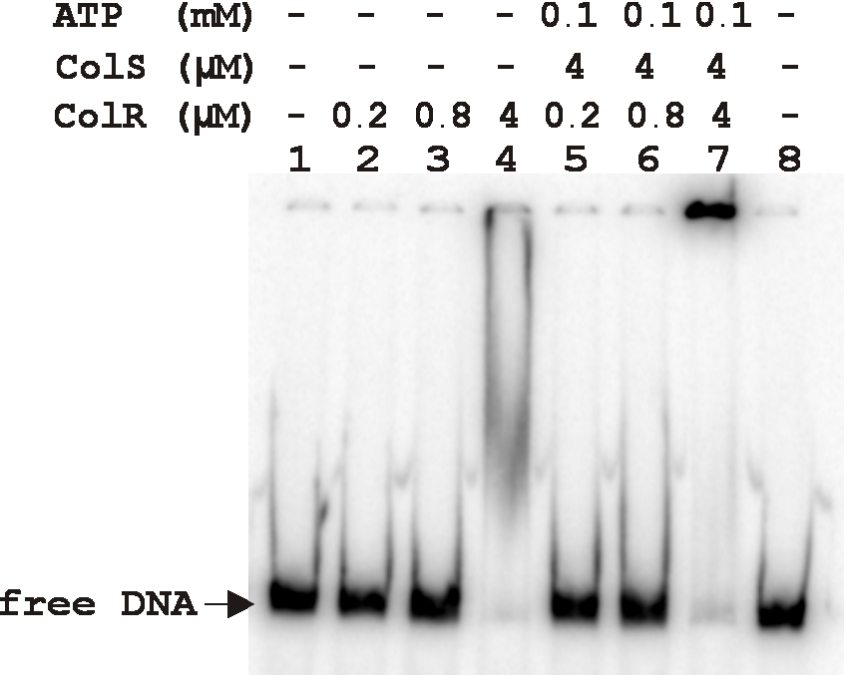
**Gel mobility shift assay of the promoter DNA of PP1636**. Approximately 0.5 ng (1000 cpm) of the DNA probe was incubated with different amounts of histidine-tagged purified ColR (lanes 2 to 4). To test the effect of phosphorylation on ColR binding activity, ColS and ATP were added to the reactions (lanes 5 to 7).

We aimed to confirm that *in silico *predicted ColR binding sites upstream of different genes, which expression was ColR-dependent *in vivo*, could really bind ColR. For that purpose, we performed a DNase I footprinting analysis to six promoter regions that were tested in a β-galactosidase assay (Fig. 3). Figure 4a shows ColR protection patterns of five different promoter regions – PP0035/0036, PP0737, PP0900/0901, PP2560/2561 and PP3766. The length of ColR-protected area varied between the promoters analysed ranging from 18-bp in one strand of PP0737 up to 46-bp in PP3766 (Fig. 4b). Phosphorylation of ColR by ColS significantly increased its affinity to DNAs upstream of PP0900/0901, PP2560/2561 and PP3766. The binding of ColR to several promoter DNAs changed the DNase I digestion pattern revealing new hypersensitive sites for DNase. Paradoxically, ColR could not affect the DNase I digestion pattern of the PP1636 promoter (data not shown), although, this promoter was influenced by ColR *in vivo *(Fig. 3) and ColR binding to this DNA was clearly detectable in a gel mobility shift assay (Fig. 5). It is noteworthy that the same was seen in case of ColR-regulated *algD *promoter (see above). Thus, performed footprint analysis could not reveal all ColR binding sites in the promoters of its target genes. Nevertheless, data presented in Fig. 4 verifies that ColR indeed binds to the computationally predicted sites in five new ColR-responsive promoters.

### ColR binding consensus and potential regulon

**Figure 6:**
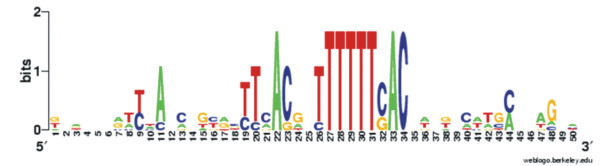
**ColR binding consensus**. Response regulator ColR binding consensus sequence created with WebLogo server (16). Sequence logo was drawn from 7 different ColR binding sites (Table 1) identified upstream of PP0035/0036, *oprQ *(PP0268), PP0737, PP0900, PP0903, PP2560/2561, and PP3766. The degree of sequence conservation at each position is indicated as a total height of a stack of letters, measured in arbitrary "bit" units, with two bits possible at each position.

The sequences of seven ColR binding sites identified by DNase I footprinting analysis (Fig. 2 and Fig. 4) were aligned to define a more precise ColR binding consensus than initial one, which was deduced only from two binding motifs detected in the promoters of *oprQ *and PP0903. The length of the region protected by ColR varied significantly in different promoters (Fig. 2 and Fig. 4). Therefore, rather long DNA fragments (presented in Table 1) were compared in order to find out whether sequences surrounding the core ColR-recognition site are conserved as well. Fig. 6 demonstrates a ColR binding consensus created using WebLogo server [16]. In the middle of the sequence a well-conserved 16-bp-long core with 8 totally conserved positions is present, whereas most of the distant positions are much less conserved. Therefore, only the core region was defined as a final ColR binding consensus ((T/C)(T/C)NA(C/G)NN(T/C)TTTTT(C/G)AC). Next, this 16-bp-long core was used to predict minimal regulon of ColRS system. 41 ColR binding motif-resembling sites were extracted from *P. putida *genome by searching the upstream regions (500 bp upstream of the translation start) of all genes using a PredictRegulon server tool. As several binding sites locate between divergently transcribed genes one may assume that ColR regulon potentially consists of more than 50 different genes (see Additional file 1). However, whilst the first 11 sites of prediction match perfectly with 7 input sequences, the rest of the matches score lower than the cut-off value (6.76929 in our prediction; see Additional file 1). Therefore, they should be considered as potential novel ColR sites, which should be confirmed experimentally. It is notable that three genes (PP1692, PP2322 and PP2323) predicted as ColR-regulated possess two potential ColR-binding sites in their promoter regions (Additional file 1). There are genes of different functions in the predicted regulon of ColRS two-component system (Additional file 1), but in accordance with previous results, this signal system seems to regulate many membrane associated functions. Namely, 56% of putative ColR binding sites locate upstream of genes implicated in various membrane functions. Furthermore, inspection of the first 26 sites shows that even 87% of them locate upstream of genes coding for membrane proteins (Additional file 1). It is also remarkable that about one third of the putative members of ColR regulon code for hypothetical proteins (Additional file 1).

**Table 1:** Seven sequences that were used for creating ColR binding consensus

**Sequence^a^**	**Locus/Gene**	**Product**
ACGATAGTTCAGCCTTTT**TTCACGTTTTTTTCAC**AACGCAAAGCTTTGCA	PP0737	conserved hypothetical protein

TGAAATGCCCACATAGGC**TTCACACTTTTTTCAC**AAGGACTGGCTCAGTA	PP3766/gloA	lactoylglutathione lyase

TTACGCATTTACATTTAG**TTCACTATTTTTTCAC**CTTTGCCTCCATAGGC	PP0035	GtrA family protein

GGGGAAAATTCACCGCAC**TTGACGGTTTTTTCAC**TGATCCTTCACGGACA	PP0900	PAP2 family protein

GCATCGCATTATCAGCGG**CTAAGATTTTTTTGAC**GAAATATTGACAAGCT	PP2561	secreted hemolysin-type calcium-binding bacteriocin, putative

GGTTATATCAACCTGCAT**TTCACGACTTTTTGAC**ATTTGTAATCGCACAC	PP0267/oprQ	outer membrane protein OprE3

TATCAGGTCAACGGGTGC**CCAACGTTTTTTTCAC**ATTGGCATGACCTGAT	PP0903	conserved hypothetical protein

^a ^Most conserved 16-bp-long sequences that were used as input in the final regulon prediction are indicated in bold

## Discussion

Two-component signal transduction systems reside at the top of regulatory cascades. Therefore, to decipher the role of a particular two-component pathway it is crucial to specify the downstream components of the cascade. This study focuses on identification of target genes of a *Pseudomonas *two-component system ColRS, thus serving as an indispensable step in the way of unravelling the mechanisms that trigger different phenotypes related to ColRS deficiency.

Previously, we have searched for ColR-regulated genes by using a promoter library created from total chromosomal DNA of *P. putida *[8]. This screen disclosed only two ColR target genes, thus forcing us to try different approach to acquire more information about the putative regulon of ColRS system. We aimed to define a binding consensus for the transcription factor ColR and to search for similar sequences in the upstream regions of genes in the genome of *P. putida*. Seven experimentally verified ColR binding sites demonstrated a highly conserved ColR-binding motif with 8 fully conserved nucleotides in the 16-bp-long core binding box (Fig. 6). The binding motifs of OmpR subfamily response regulators, among whom ColR belongs , consist typically of direct repeats separated by four to five nucleotides [17]. Careful inspection of ColR binding sites reveals that there are also two direct repeats. Although one of the repeats is much less conserved than the other, such structure of the binding site indicates that ColR binds to the DNA as a dimer. Notably, our data suggest that in addition to the highly conserved core sequence, ColR may also recognize and bind to less conserved sites. Namely, ColR regulates *algD *promoter *in vivo *and binds to this promoter in a phosphorylation-dependent manner according to gel mobility shift experiments [8]. Yet, computational analysis could not find a good ColR binding motif and DNase I protection analysis did not locate ColR binding site in the promoter region of *algD*. Nevertheless, the sequence upstream of *algD *contains many T-rich tracks characteristic for ColR binding consensus. If we lowered the specificity of Virtual Footprint prediction by allowing three mismatches in the input consensus then four putative ColR binding sites were found in *algD *promoter region, which was previously shown to bind ColR in DNA shift assay (data not shown). Probably ColR binds to these less conserved and thereby with lower affinity sites, but DNA cleavage by DNase I destabilizes the ColR-DNA complex and hence the ColR protected area cannot be detected by DNase footprint assay. Analogously, the nucleoprotein complex between the ColR and the upstream region of PP1636 probably dissociates due to DNase I cleavage-caused destabilization.

Given that some promoter regions (e.g. those of PP1692 and PP2322/2323) contain putatively more than one ColR consensus-resembling site (Additional file 1) one may hypothesise that co-operative binding of ColR to several sites may be necessary for regulation of these ColR target promoters. In this connection it is interesting to note that *in silico *analysis of seven experimentally verified ColR-binding promoters, revealed more putative ColR boxes than identified by DNase footprint assay. Namely, promoter regions of *oprQ*, PP0900/0901 and PP2560/2561 contain additional less-conserved ColR boxes (data not shown). Further experiments should prove whether these additional sites really support ColR binding to these promoters.

In this study several new ColR-dependent promoters were identified. Intriguingly, the effect of ColR absence upon promoter activities was rather modest (PP0035, PP0900, PP0737, PP1636) or not detectable (PP0036, PP2560, PP3766) when bacteria grew on glucose medium (Fig. 3). The greatest effect was seen in case of PP0903 promoter whose activity was 10-fold down-regulated in a ColR-deficient *P. putida *(Fig. 1). However, the role of ColR in regulation of its target genes was confirmed by analysis of promoter activities under conditions of ColR over-expression. Indeed, most promoters tested were strongly influenced by ColR over-expression, especially when phenol was present in the growth medium (Fig. 1 and Fig. 3). This data indicate that the amount of transcriptionally active ColR is not sufficient enough to influence significantly most of its target promoters in wild-type *P. putida *in our assay conditions. Given that the phosphorylated form of ColR bound to its recognition sequences more avidly it is reasonable to conclude that actually the phosphorylated form of ColR is limiting in bacteria growing on glucose solid medium. Therefore, we consider that most probably the ColS-activating signal is low under our assay conditions. ColR over-expression could apparently mimic the conditions evoked by the signal and therefore the ColR-dependence of its target promoters was seen more clearly in the situation of ColR over-expression. This scenario is in good accordance with our previous data showing that over-expression of ColR can compensate the defect in signal transfer from ColS to ColR when the participation of ColRS system in regulation of Tn*4652 *transposition was examined [7]. The signal sensed by ColS is not known so far. However, our observation that the impact of ColR on the expression of its target promoters was greater in the presence of phenol (Fig. 1 and Fig. 3) indicates that phenol-caused stress could be one of the conditions where ColRS signal transduction pathway is, at least partially, activated. This suggestion agrees with our previous results demonstrating the participation of ColRS system in phenol tolerance of *P. putida *[8].

Current study shows that ColR regulates directly genes locating upstream and downstream of its own gene. Like in *P. fluorescens*, ColR of *P. putida *also activates the downstream of *colRS *locating operon, that was suggested to be involved in fine-tuning of the outer membrane permeability [11]. It is notable that ColR regulates several other outer membrane protein-encoding genes confirming the role of ColRS system in the regulation of membrane functionality [8,9,11]. In addition to our previous finding that outer membrane protein-encoding *oprQ *is negatively controlled by ColR [8], the current study revealed that ColR is a negative factor also for PP0737 which codes for a protein orthologous to lipid A 3-O-deacylase PagL in *P. aeruginosa *[5]. The outer membrane-locating PagL modifies lipopolysaccharides by deacylation of lipid A at 3-O-position [18,19]. PagL becomes important under specific conditions only, e.g., it will be needed for mutants deficient in aminoarabinose-modified lipid A to resist cationic antimicrobial peptides [20]. In pathogenic Gram-negative bacteria the PagL-dependent deacylation of lipid A reduces the ability of lipid A to activate the Toll-like receptor 4 of the host, thus helping pathogens to avoid innate immune recognition [21]. The role of PagL has not been studied in *P. putida *so far.

Our data show that besides affecting the composition of outer membrane, ColR also regulates other membrane compartments. For instance, ColR activated PP0900 and PP1636 coding for two cytoplasmic membrane-locating enzymes, putative type 2 phosphatidic acid phosphatase (PAP2) and diacylglycerol kinase DgkA, respectively. These two enzymes most probably affect the membrane lipid homeostasis as they reversely regulate the abundance of phosphatidic acid and diacylglycerol, the precursor of phospholipid synthesis and the by-product of the synthesis of membrane-derived oligosaccharides, respectively [22]. Given that prediction of ColR regulon revealed another fatty acid and phospholipid metabolism related gene *acpP *(PP1915), coding for acyl carrier protein, as a putative ColR target gene, indeed, ColRS system may be involved in phospholipids homeostasis.

Since ColR is highly conserved among all *Pseudomonas *species [5] it was reasonable to presume that ColR-binding sites may also be similar in pseudomonads. Genome-wide prediction of potential ColR-binding sites in *P. fluorescens *PfO-1 disclosed seven genes that could be members of ColR regulon both in *P. putida *and *P. fluorescens *(see Additional file 2). Namely, ColR-binding box was found upstream of *P. fluorescens *PfO-1 genes orthologous to *P. putida dgkA-1*, *colR*/PP0900, PP0737, PP0903, PP1058, PP1692 and PP5152 (data not shown). Additional screening of *P. aeruginosa *PAO1, *P. syringae tomato *DC3000 and *P. syringae phaseolicola *1448A revealed that all these organisms contain perfect ColR-recognition sites upstream of *dgkA-1 *orthologs. Therefore, it is highly possible that *dgkA *is a member of ColR regulon in all these pseudomonads.

## Conclusion

Current study identified a 16-bp-long binding consensus of response regulator ColR ((T/C)(T/C)NA(C/G)NN(T/C)TTTTT(C/G)AC), which helped us to discover new genes controlled by ColRS two-component system in *P. putida*. Notably, several new ColR target genes (PP0035, PP0737, PP0900, PP1636 and PP2560) code for different membrane proteins supporting our previous assumption that the primary target of ColRS two-component system is the cell membrane. Regulon prediction suggests that ColR could regulate over 40 genes and many of them code for membrane-associated functions as well. However, it is not clear yet which of the target genes are responsible for specific ColR-related phenotypes such as lowered phenol tolerance, hindrance of transposition of Tn*4652 *and glucose-induced lysis of a subpopulation of the *colR *mutant [7-9]. Considering the number of ColR regulon genes it is highly possible that not one but several ColR target genes are involved in the formation of above-mentioned phenotypes. Given that ColRS two-component system is regulating several membrane-related genes, our further experiments are directed towards clarification the role of ColR target genes in the membrane functionality.

## Methods

### Bacterial strains, plasmids and media

*P. putida *strain PaW85 [23], which is isogenic to fully sequenced KT2440 [24], its *colR-*deficient derivative PaWcolR [7] and PaWcolR derivative strain PaWRtaccolR capable of over-expression of ColR [7] were used in this study. *E. coli *strain DH5α (Invitrogen) was used for DNA manipulations. Bacteria were grown in Luria-Bertani (LB) medium [25] or in M9 minimal medium [26] containing 10 mM glucose. Concentration of phenol was 2.5 mM in minimal medium. When necessary, the growth medium was supplemented with ampicillin (100 μg ml^-1^) or chloramphenicol (20 μg ml^-1^) for *Escherichia coli *and with benzylpenicillin (1,500 μg ml^-1^), chloramphenicol (300 μg ml^-1^) or kanamycin (50 μg ml^-1^) for *P. putida*. X-gal (5-bromo-4-chloro-3-indolyl-beta-D-galactopyranoside) (75 μg ml^-1^) was added to the growth medium for visual evaluation of promoter activities. 0.5 mM IPTG (isopropyl β-D-1-thiogalactopyranoside) was used to induce P_tac _promoter. *P. putida *was incubated at 30°C and *E. coli *at 37°C. *E. coli *and *P. putida *cells were electrotransformed according to the protocol of Sharma and Schimke [27].

### Construction of reporter plasmids

For cloning of different promoter regions into promoter probe plasmid p9TT_B_lacZ [8] the PCR-amplified DNA fragments were used. Restriction site-containing oligonucleotides used in the process are listed in Additional file 3. Different promoter regions were amplified by PCR using the purified chromosomal DNA of *P. putida *PaW85 as a template. PCR fragments were restricted with the appropriate enzyme (Additional file 3) and cloned upstream of *lacZ *gene in the plasmid p9TT_B_lacZ. Orientation of a promoter fragment was verified by PCR.

### Enzyme assay

All enzyme activities presented in this paper were measured from solid-medium-grown bacteria. Bacteria grown both on glucose or glucose plus 2.5 mM phenol containing M9 minimal medium were scraped off from the plates using toothpicks and suspended in M9 solution. For one suspension 24-hours-grown bacteria were collected from a sector comprising approximately one-twelfth of the Petri plate. β-galactosidase activity was assayed according to a previously described protocol [28].

### DNA gel mobility shift assay

ColR and N-terminally truncated ColS used in DNA gel mobility shift assay, were over-expressed and purified as His-tagged proteins by published protocol [7]. Oligonucleotides used in PCR to generate DNA probes are listed in Additional file 3. Gel mobility shift assay was performed according to a previously described protocol [8].

### DNase I footprinting assay

DNA fragments for DNase I footprinting assay were amplified from the purified chromosomal DNA of *P. putida *PaW85 by PCR. Oligonucleotides used to generate DNA probes by PCR are listed in Additional file 3. One oligonucleotide was end-labelled by phosphorylation with [α-^32^P]-ATP and thus PCR reactions created products with specific labelling of one DNA strand. The labelled DNA fragments were purified by native 5% polyacrylamide gel electrophoresis, eluted (buffer containing 0.5 M NH_4_Ac, 10 mM MgAc, 1 mM EDTA and 0.1% SDS) and re-suspended in water. For the binding reaction, different amounts of purified *P. putida *his-tagged ColR protein (concentrations of ColR are specified in the text as they varied in case of different DNA probes) were combined with 30 000 c.p.m. of labelled DNA fragment, 25 mM Tris-HCl (pH 7.5), 10 mM MgCl_2_, 1 mM CaCl_2_, 0.1 mM EDTA, 50 mM KCl, 5 μg of BSA, 5 μg of salmon sperm DNA and 5% glycerol in a final volume of 100 μl. Reactions with different volume of proteins were equalized with the addition of appropriate amount of ColR storage buffer. To test the binding of phosphorylated ColR with the DNA probes, ColS was first autophosphorylated by incubation in the presence of 0.1 mM ATP in the reaction buffer for 15 min. After addition of ColR to the phosphorylated ColS and further incubation for 15 min, labelled DNA was added to the reaction mixture. ColR was allowed to bind to DNA during 20 min at room temperature before the start of digestion by DNase I (0.25 U, Fermentas) for 3 min. Reactions were stopped by the addition of 100 μl of a solution containing 0.1 M EDTA, 0.1% sodium dodecyl sulphate, 1.6 M ammonium acetate and 20 μg of sonicated salmon sperm DNA per ml. The footprinting reaction mixtures were subsequently extracted once with phenol and chloroform (1:1 v/v) and once with chloroform and, finally, the DNA was precipitated with ethanol. The DNA fragments were resuspended in 7 μl of sequence loading buffer (deionized formamide containing 10 mM EDTA, 0.3% bromophenol blue and 0.3% xylene cyanol) and loaded onto a 6.5% polyacrylamide gel that contained 8 M urea. DNA sequencing reactions were performed with a Sequenase version 2.0 kit (US Biochemicals) and were loaded on a sequencing gel as size markers. After the run, the gels were dried and exposed to a PhosphorImager screen (Amersham Biosciences).

### *In silico *identification of putative ColR binding sites

Putative ColR binding sites in the genomes of *P. putida *KT2440,*P. aeruginosa *PAO1, *P. fluorescens *PfO-1, *P. syringae tomato *DC3000 and *P. syringae phaseolicola *1448A were searched using two programs: the PredictRegulon server [13] and the Virtual Footprint server [15]. Variations were made in the input sequence length and strand orientation. In Virtual Footprint predictions also different number of mismatches from the ColR binding consensus IUPAC code was allowed. Except for the parameters mentioned above, the programs were used with default settings. The new binding sites of *P. putida *ColR presented in this study were identified in a step-by-step process meaning that new predictions were made with every additional confirmed ColR binding site. Namely, first prediction with two input sequences (ColR sites in promoters of *oprQ *and PP0903) disclosed potential ColR binding sites in upstream regions of PP1636 and between divergently located PP0900 and PP0901 (*colR*). After experimental verification of ColR site between PP0900 and PP0901, the second round of prediction was performed with three input sequences resulting, for instance, in prediction of potential ColR sites upstream of PP0737 and PP0035. After verification of these sites, the third prediction was performed etc. Following such step-by-step process we were able to map seven ColR recognition sites, which were used as input in final prediction presented in Additional file 1.

## Authors' contributions

PAK carried out *in vitro *binding experiments and most enzyme activity measurements, participated in the *in silico *analysis and drafted the first version of the manuscript. RK and RH constructed the promoter-probe plasmids and contributed to the enzyme measurements. MK participated in manuscript editing. RH conceived, designed and coordinated experimental work and manuscript editing. All authors read and approved the final manuscript.

## Supplementary Material

Additional file 1 **Predicted regulon of ColR in *P. putida***. Output of PredictRegulon web server listing potential targets of response regulator ColR in *P. putida*. Click here for file

Additional file 2 **Potential ColR target genes in *Pseudomonas fluorescens *PfO-1**. Virtual footprint predictions with IUPAC input sequences (YYVASDNYTTTTTSAC) and (GTSAAAAARNHSTBRR) in *Pseudomonas fluorescens *PfO-1 genome. Click here for file

Additional file 3 **Oligonucleotides**. Sequences of oligonucleotides used in a current study. Click here for file
