# Supplementary material for: Identification of ColR binding consensus and prediction of regulon of ColRS two-component system
Source: BMC Mol Biol. 2009 May 16;10:46. doi: 10.1186/1471-2199-10-46 (PMC2689224; doi:10.1186/1471-2199-10-46)
Supplement: Additional file 1 — Predicted regulon of ColR in P. putida. Output of PredictRegulon web server listing potential targets of response regulator ColR in P. putida. [file 1471-2199-10-46-S1.doc]

## Additional file 1

## Supplementary table 1 - Output of PredictRegulon web server listing potential targets of response regulator ColR in *P. putida*^a^

| **Site** | **Score** | **Position from ATG** | **ColR binding site** | **Gene** | **Locus** | **Product** | **Putative**  **location** |
| --- | --- | --- | --- | --- | --- | --- | --- |
| **1** | **7.38331** | **-100** | **GTGAAAAAAACGTGAA** | **-** | **PP0737** | **conserved hypothetical protein** | **OM** |
| **2** | **7.30122** | **-207** | **TTCACACTTTTTTCAC** | **gloA** | **PP3766** | **lactoylglutathione lyase** | **CP** |
| **3** | **7.28961** | **-424** | **TTCACTATTTTTTCAC** | **-** | **PP0034** | **glycosyl transferase, group 2 family protein** | **CM** |
|  | **7.28961** | **-39** | **TTCACTATTTTTTCAC** | **-** | **PP0035** | **GtrA family protein** | **CM** |
|  | **7.28961** | **-235** | **GTGAAAAAATAGTGAA** | **-** | **PP0036** | **transcriptional regulator, putative** | **CP** |
| **4** | **7.28738** | **-85** | **TTGACGGTTTTTTCAC** | **-** | **PP0900** | **PAP2 family protein** | **CM** |
|  | **7.28738** | **-115** | **GTGAAAAAACCGTCAA** | **colR** | **PP0901** | **DNA-binding response regulator ColR** | **CP** |
| 5 | 7.24581 | -63 | TTCACGCTTTTTTGAC | dgkA-1 | PP1636 | diacylglycerol kinase | CM |
| 6 | 7.13162 | -303 | GTGAAAAAATCGTCAG | - | PP1692 | hypothetical protein | ? |
| 7 | 6.93528 | -314 | TTCAGGCTTTTTTTAC | acnB | PP2339 | aconitate hydratase 2 | CP |
|  | 6.93528 | -72 | GTAAAAAAAGCCTGAA | - | PP2340 | conserved hypothetical protein | CP |
| **8** | **6.89723** | **-279** | **GTCAAAAAGTCGTGAA** | **-** | **PP0267** | **outer membrane ferric siderophore receptor, putative** | **OM** |
|  | **6.89723** | **-212** | **TTCACGACTTTTTGAC** | **oprQ** | **PP0268** | **outer membrane protein OprE3** | **OM** |
| 9 | 6.84619 | -94 | CTGACCATTTTTTAAC | - | PP0592 | oxidoreductase, short chain dehydrogenase/reductase family | EC |
| **10** | **6.8052** | **-83** | **CCAACGTTTTTTTCAC** | **-** | **PP0903** | **conserved hypothetical protein** | **CP** |
| **11** | **6.76929** | **-260** | **GTCAAAAAAATCTTAG** | **-** | **PP2560** | **transport protein HasD, putative** | **CM** |
|  | **6.76929** | **-267** | **CTAAGATTTTTTTGAC** | **-** | **PP2561** | **secreted hemolysin-type calcium-binding bacteriocin, putative** | **EC** |
| 12 | 6.74416 | -178 | GTAAAAAAAGAGTCAT | acpP | PP1915 | acyl carrier protein | CP |
| 13 | 6.7413 | -328 | GTTAAAAAAGGCTAAA | oprI | PP2322 | outer membrane lipoprotein OprI | OM |
|  | 6.7413 | -46 | TTTAGCCTTTTTTAAC | - | PP2323 | conserved domain protein | CP |
| 14 | 6.72395 | -67 | TTTACCGATTTTTCAC | - | PP5152 | conserved hypothetical protein | ? |
| 15 | 6.7218 | -73 | TTTACGCCTTTTTTAC | - | PP3953 | potassium uptake protein, TrkH family | CM |
| 16 | 6.65622 | -180 | TGCAGATTTTTTTCAC | oprI | PP2322 | outer membrane lipoprotein OprI | OM |
|  | 6.65622 | -194 | GTGAAAAAAATCTGCA | - | PP2323 | conserved domain protein | CP |
| 17 | 6.63284 | -29 | TTCAGGTTTTTTTCAG | - | PP2138 | conserved hypothetical protein | ? |
|  | 6.63284 | -381 | TTCAGGTTTTTTTCAG | topA | PP2139 | DNA topoisomerase I | CP |
| 18 | 6.62084 | -80 | CTTACAAATTTTTCAC | - | PP1058 | conserved hypothetical protein | OM |
|  | 6.62084 | -328 | GTGAAAAATTTGTAAG | - | PP1059 | amino acid permease | CM |
| 19 | 6.60096 | -90 | TTGACGTTATTTTCAC | - | PP2579 | membrane protein, putative | CM |
|  | 6.60096 | -138 | GTGAAAATAACGTCAA | - | PP2580 | conserved hypothetical protein | ? |
| 20 | 6.58246 | -1 | GTGAAAAAACTCTTCA | - | PP4305 | periplasmic thiosulfate-binding protein | PP |
| 21 | 6.5483 | -95 | TTGACATTTTCTTCAC | rpoH | PP5108 | RNA polymerase sigma-32 factor | CP |
| 22 | 6.53691 | -119 | TTGAAAAAACCCTCAA | fliC | PP4378 | flagellin FliC | EC |
| 23 | 6.5326 | -328 | TCGATCCTTTTTTCAC | - | PP2946 | peptidyl-tRNA hydrolase domain protein | ? |
| 24 | 6.52997 | -115 | TTCACGCTTTCTTGAC | - | PP4057 | outer membrane autotransporter barrel domain protein | OM |
| 25 | 6.49289 | -86 | GTCAAAAAAACGACAA | - | PP2628 | ABC transporter, ATP-binding protein | CM |
| 26 | 6.48416 | -339 | GTGAAAAAACGGTTTT | mqo-1 | PP0751 | malate:quinone oxidoreductase | ? |
|  | 6.48416 | -267 | AAAACCGTTTTTTCAC | - | PP0752 | hypothetical protein | ? |
| 27 | 6.47935 | -172 | TAAATGGTTTTTTGAC | - | PP4086 | conserved domain protein | CP |
|  | 6.47935 | -45 | GTCAAAAAACCATTTA | - | PP4087 | hypothetical protein | ? |
| 28 | 6.44269 | -73 | TTCAGAGTTTTTTGAA | - | PP3260 | DNA ligase, ATP-dependent, putative | ? |
|  | 6.44269 | -134 | TTCAAAAAACTCTGAA | - | PP3261 | conserved hypothetical protein | CP |
| 29 | 6.43965 | -89 | TTGAATTTTTTTTCAA | - | PP1692 | hypothetical protein | ? |
| 30 | 6.41081 | -498 | TTGACACTGTTTTGAC | - | PP0676 | transcription elongation factor, putative | CP |
|  | 6.41081 | -74 | TTGACACTGTTTTGAC | - | PP0677 | lipoprotein, putative | ? |
| 31 | 6.4073 | -301 | TGAATGGTTTTTTTAC | - | PP2828 | hypothetical protein | ? |
| 32 | 6.39533 | -245 | GTCAGGCGTTTTTCAC | - | PP3536 | hypothetical protein | ? |
| 33 | 6.36925 | -82 | TTTACGCTTTTTTATC | - | PP4538 | acyl carrier protein phosphodiesterase, putative | ? |
|  | 6.36925 | -409 | GATAAAAAAGCGTAAA | - | PP4539 | transcriptional regulator, LysR family | CP |
| 34 | 6.33453 | -111 | GTAAAAAATCTGTCAC | - | PP0541 | acetyltransferase, GNAT family | CP |
| 35 | 6.33356 | -322 | TTTAAAAAAATCTCAA | - | PP1819 | methyl-accepting chemotaxis transducer | CM |
| 36 | 6.32715 | -134 | GTAAAAAAATAGCCAA | - | PP3914 | hypothetical protein | CP |
| 37 | 6.32638 | 12 | TTCAAAAAACAGTTAT | - | PP1259 | amino acid transporter, putative | CM |
| 38 | 6.32563 | -145 | GTAAAAAAGAGCTGAG | - | PP3676 | hypothetical protein | ? |
| 39 | 6.32429 | -168 | GTGAAACAATCGTTAT | - | PP1487 | conserved hypothetical protein | ? |
| 40 | 6.30888 | -81 | GTCAAAAAAGCATTTG | - | PP2986 | oxidoreductase, putative | CP |
| 41 | 6.25993 | -127 | ATCAAAAAATTGTGTA | - | PP4285 | transthyretin family protein | ? |

^a^ Results of two predictions with different strand orientation are compiled. Sequences in bold represent the experimentally confirmed ColR binding sites used as input in PredictRegulon analysis. Putative location of the protein is presented in the last column of the table (EC – extracytoplasmic, OM – outer membrane, PP – periplasm, CM – cytoplasmic membrane, CP – cytoplasmic, ? – unknown).
