# Supplementary material for: Identification of ColR binding consensus and prediction of regulon of ColRS two-component system
Source: BMC Mol Biol. 2009 May 16;10:46. doi: 10.1186/1471-2199-10-46 (PMC2689224; doi:10.1186/1471-2199-10-46)
Supplement: Additional file 2 — Potential ColR target genes in Pseudomonas fluorescens PfO-1. Virtual footprint predictions with IUPAC input sequences (YYVASDNYTTTTTSAC) and (GTSAAAAARNHSTBRR) in Pseudomonas fluorescens PfO-1 genome. [file 1471-2199-10-46-S2.doc]

**Additional file 2**

**Supplementary table 2.** Virtual footprint prediction with IUPAC input sequence (YYVASDNYTTTTTSAC) and 1 allowed mismatch on *Pseudomonas fluorescens* strain PfO-1 genome

| Number of matches: 63 |
| --- |
| Number of genes: 43 |

| Genome Browser | Start | End | Strand | PWM Score(s) | SEP Score | Sequence | ORF ID | Gene Name/Acc | ATG-Distance | Location | SMILE Analysis |
| --- | --- | --- | --- | --- | --- | --- | --- | --- | --- | --- | --- |
| [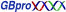](http://www.prodoric.de/gbpro.php?spos=16161&epos=20176&replicon=Pseudomonas%20fluorescens%20%28strain%20PfO-1%29&default=1) | 18161 | 18176 | - | 1.00 | -7.98 | CTGAAAAAGCCGTGGG | - | - | - | coding region in gene Pfl_0015 (Pfl_0015) | - |
| [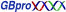](http://www.prodoric.de/gbpro.php?spos=118876&epos=122891&replicon=Pseudomonas%20fluorescens%20%28strain%20PfO-1%29&default=1) | 120876 | 120891 | - | 1.00 | -6.80 | GCCAAAAAACACTCAA | - | - | - | non-coding region between gene Pfl_0118 (Pfl_0118) and Pfl_0119 (Pfl_0119) | - |
| [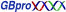](http://www.prodoric.de/gbpro.php?spos=255206&epos=259221&replicon=Pseudomonas%20fluorescens%20%28strain%20PfO-1%29&default=1) | 257206 | 257221 | - | 1.00 | -5.84 | GTCAAAAAACACGCAG | Pfl_0215 | [Pfl_0215 (GE00836329)](http://www.prodoric.de/gene.php?gene_acc=GE00836329) | 137 | intergenic | [SMILE](http://www.prodoric.de/vfp/smile.php?pattern_no=1237391928&gene_acc=GE00836329) |
| [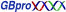](http://www.prodoric.de/gbpro.php?spos=255206&epos=259221&replicon=Pseudomonas%20fluorescens%20%28strain%20PfO-1%29&default=1) | 257206 | 257221 | - | 1.00 | -5.84 | GTCAAAAAACACGCAG | Pfl_0216 | [Pfl_0216 (GE00836330)](http://www.prodoric.de/gene.php?gene_acc=GE00836330) | 14 | intergenic | [SMILE](http://www.prodoric.de/vfp/smile.php?pattern_no=1237391928&gene_acc=GE00836330) |
| [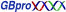](http://www.prodoric.de/gbpro.php?spos=423376&epos=427391&replicon=Pseudomonas%20fluorescens%20%28strain%20PfO-1%29&default=1) | 425376 | 425391 | - | 1.00 | -7.04 | GTCGAAAAGCCCTTAA | Pfl_0373 | [Pfl_0373 (GE00836489)](http://www.prodoric.de/gene.php?gene_acc=GE00836489) | 287 | intergenic | [SMILE](http://www.prodoric.de/vfp/smile.php?pattern_no=1237391928&gene_acc=GE00836489) |
| [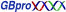](http://www.prodoric.de/gbpro.php?spos=484929&epos=488944&replicon=Pseudomonas%20fluorescens%20%28strain%20PfO-1%29&default=1) | 486929 | 486944 | - | 1.00 | -7.76 | GTGAAAAAATCCTCAT | - | - | - | coding region in gene Pfl_0428 (Pfl_0428) | - |
| [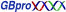](http://www.prodoric.de/gbpro.php?spos=578592&epos=582607&replicon=Pseudomonas%20fluorescens%20%28strain%20PfO-1%29&default=1) | 580592 | 580607 | - | 1.00 | -7.66 | CTGAAAAAGCTCTGGG | - | - | - | coding region in gene Pfl_0501 (Pfl_0501) | - |
| [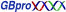](http://www.prodoric.de/gbpro.php?spos=665863&epos=669878&replicon=Pseudomonas%20fluorescens%20%28strain%20PfO-1%29&default=1) | 667863 | 667878 | - | 1.00 | -7.71 | GTGAAGAAGGTGTTGA | - | - | - | coding region in gene Pfl_0575 (Pfl_0575) | - |
| [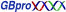](http://www.prodoric.de/gbpro.php?spos=680044&epos=684059&replicon=Pseudomonas%20fluorescens%20%28strain%20PfO-1%29&default=1) | 682044 | 682059 | + | 1.00 | -7.57 | CCGAGAATTTTTTGAT | - | - | - | coding region in gene Pfl_0590 (Pfl_0590) | - |
| [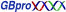](http://www.prodoric.de/gbpro.php?spos=711295&epos=715310&replicon=Pseudomonas%20fluorescens%20%28strain%20PfO-1%29&default=1) | 713295 | 713310 | - | 1.00 | -7.64 | GTGGAAAAGAACTGGA | - | - | - | coding region in gene Pfl_0609 (Pfl_0609) | - |
| [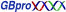](http://www.prodoric.de/gbpro.php?spos=800446&epos=804461&replicon=Pseudomonas%20fluorescens%20%28strain%20PfO-1%29&default=1) | 802446 | 802461 | - | 1.00 | -7.16 | GTCAAAAAAGCCCTGA | - | - | - | non-coding region between gene Pfl_0686 (Pfl_0686) and Pfl_0687 (Pfl_0687) | - |
| [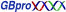](http://www.prodoric.de/gbpro.php?spos=855339&epos=859354&replicon=Pseudomonas%20fluorescens%20%28strain%20PfO-1%29&default=1) | 857339 | 857354 | - | 1.00 | -7.13 | GTGAAAAAAGTCTTGT | Pfl_0728 | [Pfl_0728 (GE00836851)](http://www.prodoric.de/gene.php?gene_acc=GE00836851) | 156 | intergenic | [SMILE](http://www.prodoric.de/vfp/smile.php?pattern_no=1237391928&gene_acc=GE00836851) |
| [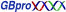](http://www.prodoric.de/gbpro.php?spos=855339&epos=859354&replicon=Pseudomonas%20fluorescens%20%28strain%20PfO-1%29&default=1) | 857339 | 857354 | - | 1.00 | -7.13 | GTGAAAAAAGTCTTGT | Pfl_0729 | [Pfl_0729 (GE00836852)](http://www.prodoric.de/gene.php?gene_acc=GE00836852) | 74 | intergenic | [SMILE](http://www.prodoric.de/vfp/smile.php?pattern_no=1237391928&gene_acc=GE00836852) |
| [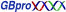](http://www.prodoric.de/gbpro.php?spos=938310&epos=942325&replicon=Pseudomonas%20fluorescens%20%28strain%20PfO-1%29&default=1) | 940310 | 940325 | - | 1.00 | -8.17 | GGCAAAAAAGTCTCGA | - | - | - | coding region in gene Pfl_0797 (Pfl_0797) | - |
| [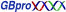](http://www.prodoric.de/gbpro.php?spos=983325&epos=987340&replicon=Pseudomonas%20fluorescens%20%28strain%20PfO-1%29&default=1) | 985325 | 985340 | - | 1.00 | -8.09 | GTGAAAAACCCGTGGA | - | - | - | coding region in gene Pfl_0836 (Pfl_0836) | - |
| [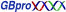](http://www.prodoric.de/gbpro.php?spos=1054263&epos=1058278&replicon=Pseudomonas%20fluorescens%20%28strain%20PfO-1%29&default=1) | 1056263 | 1056278 | - | 1.00 | -6.94 | GTCAAAACGTCCTCGA | - | - | - | coding region in gene Pfl_0908 (Pfl_0908) | - |
| [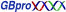](http://www.prodoric.de/gbpro.php?spos=1119678&epos=1123693&replicon=Pseudomonas%20fluorescens%20%28strain%20PfO-1%29&default=1) | 1121678 | 1121693 | - | 1.00 | -6.77 | GTCAAAAAACCCTCGC | - | - | - | non-coding region between gene Pfl_0960 (Pfl_0960) and Pfl_0961 (Pfl_0961) | - |
| [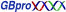](http://www.prodoric.de/gbpro.php?spos=1339103&epos=1343118&replicon=Pseudomonas%20fluorescens%20%28strain%20PfO-1%29&default=1) | 1341103 | 1341118 | + | 1.00 | -7.42 | TCGAGTTTTTTGTGAC | Pfl_1168 | [Pfl_1168 (GE00837293)](http://www.prodoric.de/gene.php?gene_acc=GE00837293) | 520 | coding region | [SMILE](http://www.prodoric.de/vfp/smile.php?pattern_no=1237391928&gene_acc=GE00837293) |
| [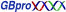](http://www.prodoric.de/gbpro.php?spos=1349788&epos=1353803&replicon=Pseudomonas%20fluorescens%20%28strain%20PfO-1%29&default=1) | 1351788 | 1351803 | - | 0.00 | -7.26 | GTCAAAAAAGCGTGAA | Pfl_1183 | [Pfl_1183 (GE00837308)](http://www.prodoric.de/gene.php?gene_acc=GE00837308) | 49 | intergenic | [SMILE](http://www.prodoric.de/vfp/smile.php?pattern_no=1237391928&gene_acc=GE00837308) |
| [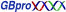](http://www.prodoric.de/gbpro.php?spos=1380061&epos=1384076&replicon=Pseudomonas%20fluorescens%20%28strain%20PfO-1%29&default=1) | 1382061 | 1382076 | - | 1.00 | -7.57 | GTGAAAAAGTCATCGA | Pfl_1209 | [Pfl_1209 (GE00837336)](http://www.prodoric.de/gene.php?gene_acc=GE00837336) | 529 | coding region | [SMILE](http://www.prodoric.de/vfp/smile.php?pattern_no=1237391928&gene_acc=GE00837336) |
| [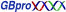](http://www.prodoric.de/gbpro.php?spos=1558072&epos=1562087&replicon=Pseudomonas%20fluorescens%20%28strain%20PfO-1%29&default=1) | 1560072 | 1560087 | - | 1.00 | -6.83 | GTGAAAAAGAAGCCAA | Pfl_1386 | [Pfl_1386 (GE00837515)](http://www.prodoric.de/gene.php?gene_acc=GE00837515) | 183 | intergenic | [SMILE](http://www.prodoric.de/vfp/smile.php?pattern_no=1237391928&gene_acc=GE00837515) |
| [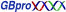](http://www.prodoric.de/gbpro.php?spos=1558072&epos=1562087&replicon=Pseudomonas%20fluorescens%20%28strain%20PfO-1%29&default=1) | 1560072 | 1560087 | - | 1.00 | -6.83 | GTGAAAAAGAAGCCAA | Pfl_1387 | [Pfl_1387 (GE00837516)](http://www.prodoric.de/gene.php?gene_acc=GE00837516) | 100 | intergenic | [SMILE](http://www.prodoric.de/vfp/smile.php?pattern_no=1237391928&gene_acc=GE00837516) |
| [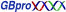](http://www.prodoric.de/gbpro.php?spos=1717592&epos=1721607&replicon=Pseudomonas%20fluorescens%20%28strain%20PfO-1%29&default=1) | 1719592 | 1719607 | - | 1.00 | -6.84 | GTCAAAAAACCGTGCA | Pfl_1532 | [Pfl_1532 (GE00837661)](http://www.prodoric.de/gene.php?gene_acc=GE00837661) | 113 | intergenic | [SMILE](http://www.prodoric.de/vfp/smile.php?pattern_no=1237391928&gene_acc=GE00837661) |
| [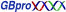](http://www.prodoric.de/gbpro.php?spos=1726464&epos=1730479&replicon=Pseudomonas%20fluorescens%20%28strain%20PfO-1%29&default=1) | 1728464 | 1728479 | - | 1.00 | -8.20 | GTCAACAAACCGTTGA | - | - | - | coding region in gene Pfl_1539 (Pfl_1539) | - |
| [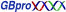](http://www.prodoric.de/gbpro.php?spos=1741972&epos=1745987&replicon=Pseudomonas%20fluorescens%20%28strain%20PfO-1%29&default=1) | 1743972 | 1743987 | - | 1.00 | -6.99 | GAGAAAAAGGCGTGGA | - | - | - | coding region in gene Pfl_1555 (Pfl_1555) | - |
| [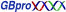](http://www.prodoric.de/gbpro.php?spos=1776251&epos=1780266&replicon=Pseudomonas%20fluorescens%20%28strain%20PfO-1%29&default=1) | 1778251 | 1778266 | - | 1.00 | -7.00 | GTGAAAAAACTCTTTG | - | - | - | coding region in gene Pfl_1591 (Pfl_1591) | - |
| [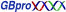](http://www.prodoric.de/gbpro.php?spos=1995359&epos=1999374&replicon=Pseudomonas%20fluorescens%20%28strain%20PfO-1%29&default=1) | 1997359 | 1997374 | + | 1.00 | -8.10 | CCGGGATCTTTTTCAC | Pfl_1793 | [Pfl_1793 (GE00837927)](http://www.prodoric.de/gene.php?gene_acc=GE00837927) | 600 | coding region | [SMILE](http://www.prodoric.de/vfp/smile.php?pattern_no=1237391928&gene_acc=GE00837927) |
| [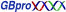](http://www.prodoric.de/gbpro.php?spos=2130971&epos=2134986&replicon=Pseudomonas%20fluorescens%20%28strain%20PfO-1%29&default=1) | 2132971 | 2132986 | - | 1.00 | -7.81 | GAGAAAAAACTGTCGG | - | - | - | coding region in gene Pfl_1870 (Pfl_1870) | - |
| [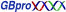](http://www.prodoric.de/gbpro.php?spos=2147823&epos=2151838&replicon=Pseudomonas%20fluorescens%20%28strain%20PfO-1%29&default=1) | 2149823 | 2149838 | - | 1.00 | -7.71 | GTGAAAATAAAGTCGA | - | - | - | coding region in gene Pfl_1885 (Pfl_1885) | - |
| [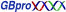](http://www.prodoric.de/gbpro.php?spos=2183251&epos=2187266&replicon=Pseudomonas%20fluorescens%20%28strain%20PfO-1%29&default=1) | 2185251 | 2185266 | - | 1.00 | -7.50 | GTCGAAAAAGTGTGGG | Pfl_1911 | [Pfl_1911 (GE00838054)](http://www.prodoric.de/gene.php?gene_acc=GE00838054) | 47 | intergenic | [SMILE](http://www.prodoric.de/vfp/smile.php?pattern_no=1237391928&gene_acc=GE00838054) |
| [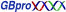](http://www.prodoric.de/gbpro.php?spos=2306828&epos=2310843&replicon=Pseudomonas%20fluorescens%20%28strain%20PfO-1%29&default=1) | 2308828 | 2308843 | - | 1.00 | -7.24 | GTGAAAAACCCGTCAA | Pfl_2024 | [Pfl_2024 (GE00838168)](http://www.prodoric.de/gene.php?gene_acc=GE00838168) | 174 | intergenic | [SMILE](http://www.prodoric.de/vfp/smile.php?pattern_no=1237391928&gene_acc=GE00838168) |
| [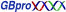](http://www.prodoric.de/gbpro.php?spos=2306828&epos=2310843&replicon=Pseudomonas%20fluorescens%20%28strain%20PfO-1%29&default=1) | 2308828 | 2308843 | - | 1.00 | -7.24 | GTGAAAAACCCGTCAA | Pfl_2025 | [Pfl_2025 (GE00838169)](http://www.prodoric.de/gene.php?gene_acc=GE00838169) | 202 | intergenic | [SMILE](http://www.prodoric.de/vfp/smile.php?pattern_no=1237391928&gene_acc=GE00838169) |
| [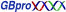](http://www.prodoric.de/gbpro.php?spos=2306920&epos=2310935&replicon=Pseudomonas%20fluorescens%20%28strain%20PfO-1%29&default=1) | 2308920 | 2308935 | - | 0.00 | -7.27 | GTCAAAAAACTGTTGA | Pfl_2024 | [Pfl_2024 (GE00838168)](http://www.prodoric.de/gene.php?gene_acc=GE00838168) | 266 | intergenic | [SMILE](http://www.prodoric.de/vfp/smile.php?pattern_no=1237391928&gene_acc=GE00838168) |
| [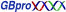](http://www.prodoric.de/gbpro.php?spos=2306920&epos=2310935&replicon=Pseudomonas%20fluorescens%20%28strain%20PfO-1%29&default=1) | 2308920 | 2308935 | - | 0.00 | -7.27 | GTCAAAAAACTGTTGA | Pfl_2025 | [Pfl_2025 (GE00838169)](http://www.prodoric.de/gene.php?gene_acc=GE00838169) | 110 | intergenic | [SMILE](http://www.prodoric.de/vfp/smile.php?pattern_no=1237391928&gene_acc=GE00838169) |
| [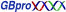](http://www.prodoric.de/gbpro.php?spos=2318031&epos=2322046&replicon=Pseudomonas%20fluorescens%20%28strain%20PfO-1%29&default=1) | 2320031 | 2320046 | - | 1.00 | -6.68 | ATGAAAAAATACTTAG | - | - | - | coding region in gene Pfl_2035 (Pfl_2035) | - |
| [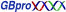](http://www.prodoric.de/gbpro.php?spos=2772539&epos=2776554&replicon=Pseudomonas%20fluorescens%20%28strain%20PfO-1%29&default=1) | 2774539 | 2774554 | - | 1.00 | -7.37 | GACAAAAAGGTGTTGA | Pfl_2417 | [Pfl_2417 (GE00838566)](http://www.prodoric.de/gene.php?gene_acc=GE00838566) | 437 | coding region | [SMILE](http://www.prodoric.de/vfp/smile.php?pattern_no=1237391928&gene_acc=GE00838566) |
| [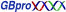](http://www.prodoric.de/gbpro.php?spos=2774507&epos=2778522&replicon=Pseudomonas%20fluorescens%20%28strain%20PfO-1%29&default=1) | 2776507 | 2776522 | + | 1.00 | -8.00 | TCGAGGATTTTTTCGC | - | - | - | coding region in gene Pfl_2420 (Pfl_2420) | - |
| [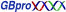](http://www.prodoric.de/gbpro.php?spos=2874670&epos=2878685&replicon=Pseudomonas%20fluorescens%20%28strain%20PfO-1%29&default=1) | 2876670 | 2876685 | - | 1.00 | -7.93 | GCCAAAAAACACTGAA | Pfl_2518 | [Pfl_2518 (GE00838668)](http://www.prodoric.de/gene.php?gene_acc=GE00838668) | 149 | coding region | [SMILE](http://www.prodoric.de/vfp/smile.php?pattern_no=1237391928&gene_acc=GE00838668) |
| [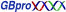](http://www.prodoric.de/gbpro.php?spos=2919095&epos=2923110&replicon=Pseudomonas%20fluorescens%20%28strain%20PfO-1%29&default=1) | 2921095 | 2921110 | - | 1.00 | -7.38 | GTCAAAAATTCGTCGA | Pfl_2549 | [glgA (GE00838699)](http://www.prodoric.de/gene.php?gene_acc=GE00838699) | 332 | intergenic | [SMILE](http://www.prodoric.de/vfp/smile.php?pattern_no=1237391928&gene_acc=GE00838699) |
| [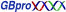](http://www.prodoric.de/gbpro.php?spos=3002872&epos=3006887&replicon=Pseudomonas%20fluorescens%20%28strain%20PfO-1%29&default=1) | 3004872 | 3004887 | - | 1.00 | -6.98 | GTGGAAAAACAGTTAA | Pfl_2627 | [Pfl_2627 (GE00838777)](http://www.prodoric.de/gene.php?gene_acc=GE00838777) | 5 | intergenic | [SMILE](http://www.prodoric.de/vfp/smile.php?pattern_no=1237391928&gene_acc=GE00838777) |
| [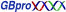](http://www.prodoric.de/gbpro.php?spos=3033770&epos=3037785&replicon=Pseudomonas%20fluorescens%20%28strain%20PfO-1%29&default=1) | 3035770 | 3035785 | - | 1.00 | -7.73 | GTCAACAAAGCCTCGA | Pfl_2649 | [Pfl_2649 (GE00838799)](http://www.prodoric.de/gene.php?gene_acc=GE00838799) | 508 | coding region | [SMILE](http://www.prodoric.de/vfp/smile.php?pattern_no=1237391928&gene_acc=GE00838799) |
| [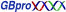](http://www.prodoric.de/gbpro.php?spos=3063391&epos=3067406&replicon=Pseudomonas%20fluorescens%20%28strain%20PfO-1%29&default=1) | 3065391 | 3065406 | - | 1.00 | -7.19 | GGGAAAAAAAACTCAG | - | - | - | coding region in gene Pfl_2668 (Pfl_2668) | - |
| [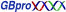](http://www.prodoric.de/gbpro.php?spos=3071972&epos=3075987&replicon=Pseudomonas%20fluorescens%20%28strain%20PfO-1%29&default=1) | 3073972 | 3073987 | - | 1.00 | -7.15 | ATCAAAAAAATCTCGG | Pfl_2673 | [Pfl_2673 (GE00838823)](http://www.prodoric.de/gene.php?gene_acc=GE00838823) | 144 | intergenic | [SMILE](http://www.prodoric.de/vfp/smile.php?pattern_no=1237391928&gene_acc=GE00838823) |
| [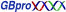](http://www.prodoric.de/gbpro.php?spos=3071972&epos=3075987&replicon=Pseudomonas%20fluorescens%20%28strain%20PfO-1%29&default=1) | 3073972 | 3073987 | - | 1.00 | -7.15 | ATCAAAAAAATCTCGG | Pfl_2674 | [Pfl_2674 (GE00838824)](http://www.prodoric.de/gene.php?gene_acc=GE00838824) | 105 | intergenic | [SMILE](http://www.prodoric.de/vfp/smile.php?pattern_no=1237391928&gene_acc=GE00838824) |
| [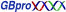](http://www.prodoric.de/gbpro.php?spos=3270773&epos=3274788&replicon=Pseudomonas%20fluorescens%20%28strain%20PfO-1%29&default=1) | 3272773 | 3272788 | - | 1.00 | -7.07 | GAGAAAAAAACGTCAA | Pfl_2837 | [Pfl_2837 (GE00838987)](http://www.prodoric.de/gene.php?gene_acc=GE00838987) | 106 | intergenic | [SMILE](http://www.prodoric.de/vfp/smile.php?pattern_no=1237391928&gene_acc=GE00838987) |
| [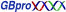](http://www.prodoric.de/gbpro.php?spos=3564989&epos=3569004&replicon=Pseudomonas%20fluorescens%20%28strain%20PfO-1%29&default=1) | 3566989 | 3567004 | + | 1.00 | -7.37 | TCCAGTTTTTTTTCCC | - | - | - | coding region in gene Pfl_3108 (Pfl_3108) | - |
| [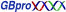](http://www.prodoric.de/gbpro.php?spos=3590750&epos=3594765&replicon=Pseudomonas%20fluorescens%20%28strain%20PfO-1%29&default=1) | 3592750 | 3592765 | - | 1.00 | -8.00 | GTGAAAAAGCTGTGAT | - | - | - | coding region in gene Pfl_3129 (Pfl_3129) | - |
| [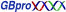](http://www.prodoric.de/gbpro.php?spos=3830495&epos=3834510&replicon=Pseudomonas%20fluorescens%20%28strain%20PfO-1%29&default=1) | 3832495 | 3832510 | - | 1.00 | -7.02 | GTCAAAAGAATGTTAG | Pfl_3357 | [Pfl_3357 (GE00839508)](http://www.prodoric.de/gene.php?gene_acc=GE00839508) | 65 | intergenic | [SMILE](http://www.prodoric.de/vfp/smile.php?pattern_no=1237391928&gene_acc=GE00839508) |
| [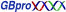](http://www.prodoric.de/gbpro.php?spos=3830495&epos=3834510&replicon=Pseudomonas%20fluorescens%20%28strain%20PfO-1%29&default=1) | 3832495 | 3832510 | - | 1.00 | -7.02 | GTCAAAAGAATGTTAG | Pfl_3358 | [Pfl_3358 (GE00839509)](http://www.prodoric.de/gene.php?gene_acc=GE00839509) | 54 | intergenic | [SMILE](http://www.prodoric.de/vfp/smile.php?pattern_no=1237391928&gene_acc=GE00839509) |
| [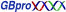](http://www.prodoric.de/gbpro.php?spos=3862276&epos=3866291&replicon=Pseudomonas%20fluorescens%20%28strain%20PfO-1%29&default=1) | 3864276 | 3864291 | - | 1.00 | -7.95 | GTGAAAACGCCCTGAA | Pfl_3388 | [Pfl_3388 (GE00839539)](http://www.prodoric.de/gene.php?gene_acc=GE00839539) | 446 | coding region | [SMILE](http://www.prodoric.de/vfp/smile.php?pattern_no=1237391928&gene_acc=GE00839539) |
| [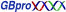](http://www.prodoric.de/gbpro.php?spos=3911587&epos=3915602&replicon=Pseudomonas%20fluorescens%20%28strain%20PfO-1%29&default=1) | 3913587 | 3913602 | - | 1.00 | -8.02 | ATGAAAAAGCTCTGGG | - | - | - | coding region in gene Pfl_3432 (Pfl_3432) | - |
| [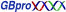](http://www.prodoric.de/gbpro.php?spos=4187918&epos=4191933&replicon=Pseudomonas%20fluorescens%20%28strain%20PfO-1%29&default=1) | 4189918 | 4189933 | - | 1.00 | -7.13 | ATGAAAAAGTCGTGAA | Pfl_3700 | [Pfl_3700 (GE00839856)](http://www.prodoric.de/gene.php?gene_acc=GE00839856) | 112 | intergenic | [SMILE](http://www.prodoric.de/vfp/smile.php?pattern_no=1237391928&gene_acc=GE00839856) |
| [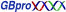](http://www.prodoric.de/gbpro.php?spos=4304547&epos=4308562&replicon=Pseudomonas%20fluorescens%20%28strain%20PfO-1%29&default=1) | 4306547 | 4306562 | - | 1.00 | -7.43 | GTCAACAAGCCCTGAG | - | - | - | coding region in gene Pfl_3804 (Pfl_3804) | - |
| [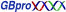](http://www.prodoric.de/gbpro.php?spos=4531904&epos=4535919&replicon=Pseudomonas%20fluorescens%20%28strain%20PfO-1%29&default=1) | 4533904 | 4533919 | - | 1.00 | -7.70 | GTGAAAAAACCGGTGG | - | - | - | coding region in gene Pfl_4006 (Pfl_4006) | - |
| [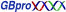](http://www.prodoric.de/gbpro.php?spos=4605776&epos=4609791&replicon=Pseudomonas%20fluorescens%20%28strain%20PfO-1%29&default=1) | 4607776 | 4607791 | + | 1.00 | -7.70 | TCGACACCTTCTTCAC | Pfl_4077 | [Pfl_4077 (GE00840237)](http://www.prodoric.de/gene.php?gene_acc=GE00840237) | 150 | coding region | [SMILE](http://www.prodoric.de/vfp/smile.php?pattern_no=1237391928&gene_acc=GE00840237) |
| [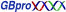](http://www.prodoric.de/gbpro.php?spos=4666349&epos=4670364&replicon=Pseudomonas%20fluorescens%20%28strain%20PfO-1%29&default=1) | 4668349 | 4668364 | - | 1.00 | -6.14 | GTGAAAAAATCATTGA | Pfl_4125 | [Pfl_4125 (GE00840285)](http://www.prodoric.de/gene.php?gene_acc=GE00840285) | 315 | coding region | [SMILE](http://www.prodoric.de/vfp/smile.php?pattern_no=1237391928&gene_acc=GE00840285) |
| [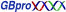](http://www.prodoric.de/gbpro.php?spos=4835243&epos=4839258&replicon=Pseudomonas%20fluorescens%20%28strain%20PfO-1%29&default=1) | 4837243 | 4837258 | - | 1.00 | -7.37 | GTGAAAAAATGCTCGG | - | - | - | coding region in gene Pfl_4286 (Pfl_4286) | - |
| [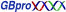](http://www.prodoric.de/gbpro.php?spos=5071957&epos=5075972&replicon=Pseudomonas%20fluorescens%20%28strain%20PfO-1%29&default=1) | 5073957 | 5073972 | - | 1.00 | -6.84 | GTGAAAAACTTGTTAA | Pfl_4498 | [Pfl_4498 (GE00840670)](http://www.prodoric.de/gene.php?gene_acc=GE00840670) | 118 | intergenic | [SMILE](http://www.prodoric.de/vfp/smile.php?pattern_no=1237391928&gene_acc=GE00840670) |
| [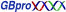](http://www.prodoric.de/gbpro.php?spos=5071957&epos=5075972&replicon=Pseudomonas%20fluorescens%20%28strain%20PfO-1%29&default=1) | 5073957 | 5073972 | - | 1.00 | -6.84 | GTGAAAAACTTGTTAA | Pfl_4499 | [Pfl_4499 (GE00840671)](http://www.prodoric.de/gene.php?gene_acc=GE00840671) | 71 | intergenic | [SMILE](http://www.prodoric.de/vfp/smile.php?pattern_no=1237391928&gene_acc=GE00840671) |
| [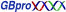](http://www.prodoric.de/gbpro.php?spos=5149237&epos=5153252&replicon=Pseudomonas%20fluorescens%20%28strain%20PfO-1%29&default=1) | 5151237 | 5151252 | - | 0.00 | -7.35 | GTGAAAAAATTGTGAA | Pfl_4562 | [Pfl_4562 (GE00840734)](http://www.prodoric.de/gene.php?gene_acc=GE00840734) | 497 | coding region | [SMILE](http://www.prodoric.de/vfp/smile.php?pattern_no=1237391928&gene_acc=GE00840734) |
| [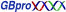](http://www.prodoric.de/gbpro.php?spos=5149237&epos=5153252&replicon=Pseudomonas%20fluorescens%20%28strain%20PfO-1%29&default=1) | 5151237 | 5151252 | - | 0.00 | -7.35 | GTGAAAAAATTGTGAA | Pfl_4564 | [Pfl_4564 (GE00840736)](http://www.prodoric.de/gene.php?gene_acc=GE00840736) | 102 | coding region | [SMILE](http://www.prodoric.de/vfp/smile.php?pattern_no=1237391928&gene_acc=GE00840736) |
| [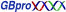](http://www.prodoric.de/gbpro.php?spos=5214255&epos=5218270&replicon=Pseudomonas%20fluorescens%20%28strain%20PfO-1%29&default=1) | 5216255 | 5216270 | - | 1.00 | -7.54 | GTCAAAAAAGACGCGA | Pfl_4617 | [Pfl_4617 (GE00840789)](http://www.prodoric.de/gene.php?gene_acc=GE00840789) | 82 | intergenic | [SMILE](http://www.prodoric.de/vfp/smile.php?pattern_no=1237391928&gene_acc=GE00840789) |
| [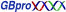](http://www.prodoric.de/gbpro.php?spos=5245801&epos=5249816&replicon=Pseudomonas%20fluorescens%20%28strain%20PfO-1%29&default=1) | 5247801 | 5247816 | - | 1.00 | -6.99 | GGGAAAAAGCCCTCGG | Pfl_4648 | [Pfl_4648 (GE00840821)](http://www.prodoric.de/gene.php?gene_acc=GE00840821) | 487 | coding region | [SMILE](http://www.prodoric.de/vfp/smile.php?pattern_no=1237391928&gene_acc=GE00840821) |
| [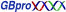](http://www.prodoric.de/gbpro.php?spos=5326953&epos=5330968&replicon=Pseudomonas%20fluorescens%20%28strain%20PfO-1%29&default=1) | 5328953 | 5328968 | - | 1.00 | -7.74 | GTGAAAAAGCCCGGGG | Pfl_4720 | [Pfl_4720 (GE00840893)](http://www.prodoric.de/gene.php?gene_acc=GE00840893) | 427 | coding region | [SMILE](http://www.prodoric.de/vfp/smile.php?pattern_no=1237391928&gene_acc=GE00840893) |
| [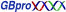](http://www.prodoric.de/gbpro.php?spos=5346756&epos=5350771&replicon=Pseudomonas%20fluorescens%20%28strain%20PfO-1%29&default=1) | 5348756 | 5348771 | + | 1.00 | -6.23 | TTCACATTTTTTTTAC | Pfl_4744 | [Pfl_4744 (GE00840917)](http://www.prodoric.de/gene.php?gene_acc=GE00840917) | 101 | coding region | [SMILE](http://www.prodoric.de/vfp/smile.php?pattern_no=1237391928&gene_acc=GE00840917) |
| [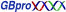](http://www.prodoric.de/gbpro.php?spos=5484368&epos=5488383&replicon=Pseudomonas%20fluorescens%20%28strain%20PfO-1%29&default=1) | 5486368 | 5486383 | - | 1.00 | -7.63 | GTCAAAAAAACGGGAG | Pfl_4868 | [Pfl_4868 (GE00841052)](http://www.prodoric.de/gene.php?gene_acc=GE00841052) | 217 | intergenic | [SMILE](http://www.prodoric.de/vfp/smile.php?pattern_no=1237391928&gene_acc=GE00841052) |
| [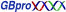](http://www.prodoric.de/gbpro.php?spos=5548612&epos=5552627&replicon=Pseudomonas%20fluorescens%20%28strain%20PfO-1%29&default=1) | 5550612 | 5550627 | - | 1.00 | -7.87 | GTGAAAAAAATCGCAG | Pfl_4924 | [Pfl_4924 (GE00841108)](http://www.prodoric.de/gene.php?gene_acc=GE00841108) | 506 | coding region | [SMILE](http://www.prodoric.de/vfp/smile.php?pattern_no=1237391928&gene_acc=GE00841108) |
| [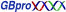](http://www.prodoric.de/gbpro.php?spos=5557168&epos=5561183&replicon=Pseudomonas%20fluorescens%20%28strain%20PfO-1%29&default=1) | 5559168 | 5559183 | - | 1.00 | -8.71 | GTGAAGAAGACGTCGA | Pfl_4934 | [Pfl_4934 (GE00841118)](http://www.prodoric.de/gene.php?gene_acc=GE00841118) | 397 | coding region | [SMILE](http://www.prodoric.de/vfp/smile.php?pattern_no=1237391928&gene_acc=GE00841118) |
| [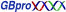](http://www.prodoric.de/gbpro.php?spos=5639013&epos=5643028&replicon=Pseudomonas%20fluorescens%20%28strain%20PfO-1%29&default=1) | 5641013 | 5641028 | - | 1.00 | -8.18 | GTGAGAAAACTGTCGA | - | - | - | coding region in gene Pfl_5004 (Pfl_5004) | - |
| [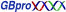](http://www.prodoric.de/gbpro.php?spos=5878630&epos=5882645&replicon=Pseudomonas%20fluorescens%20%28strain%20PfO-1%29&default=1) | 5880630 | 5880645 | - | 1.00 | -7.78 | GTGAAAAAGAAGCCGA | Pfl_5223 | [Pfl_5223 (GE00841417)](http://www.prodoric.de/gene.php?gene_acc=GE00841417) | 335 | coding region | [SMILE](http://www.prodoric.de/vfp/smile.php?pattern_no=1237391928&gene_acc=GE00841417) |
| [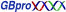](http://www.prodoric.de/gbpro.php?spos=5955691&epos=5959706&replicon=Pseudomonas%20fluorescens%20%28strain%20PfO-1%29&default=1) | 5957691 | 5957706 | - | 1.00 | -7.38 | TTGAAAAAGGTCTGGA | - | - | - | coding region in gene Pfl_5294 (Pfl_5294) | - |
| [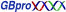](http://www.prodoric.de/gbpro.php?spos=6219804&epos=6223819&replicon=Pseudomonas%20fluorescens%20%28strain%20PfO-1%29&default=1) | 6221804 | 6221819 | - | 1.00 | -7.59 | GTCAAAAAGGCGGGAG | - | - | - | non-coding region between gene Pfl_5553 (Pfl_5553) and Pfl_5554 (Pfl_5554) |  |

**Supplementary table 3.** Virtual footprint prediction with IUPAC input sequence (GTSAAAAARNHSTBRR) and 1 allowed mismatch on *Pseudomonas fluorescens* strain PfO-1 genome

| Number of matches: 50 |
| --- |
| Number of genes: 31 |

| Genome Browser | Start | End | Strand | PWM Score(s) | SEP Score | Sequence | ORF ID | Gene Name/Acc | ATG-Distance | Location | SMILE Analysis |
| --- | --- | --- | --- | --- | --- | --- | --- | --- | --- | --- | --- |
| [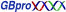](http://www.prodoric.de/gbpro.php?spos=162617&epos=166632&replicon=Pseudomonas%20fluorescens%20%28strain%20PfO-1%29&default=1) | 164617 | 164632 | - | 1.00 | -7.68 | TCAAGGCCTTGTTGAC | - | - | - | coding region in gene Pfl_0136 (Pfl_0136) | - |
| [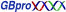](http://www.prodoric.de/gbpro.php?spos=366451&epos=370466&replicon=Pseudomonas%20fluorescens%20%28strain%20PfO-1%29&default=1) | 368451 | 368466 | - | 1.00 | -8.21 | TCGAGGCTTTTTTCGC | - | - | - | coding region in gene Pfl_0322 (Pfl_0322) | - |
| [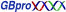](http://www.prodoric.de/gbpro.php?spos=507997&epos=512012&replicon=Pseudomonas%20fluorescens%20%28strain%20PfO-1%29&default=1) | 509997 | 510012 | - | 1.00 | -7.15 | TCCAGGGCTTTTTCAA | Pfl_0447 | [Pfl_0447 (GE00836563)](http://www.prodoric.de/gene.php?gene_acc=GE00836563) | 257 | intergenic | [SMILE](http://www.prodoric.de/vfp/smile.php?pattern_no=1237392460&gene_acc=GE00836563) |
| [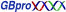](http://www.prodoric.de/gbpro.php?spos=507997&epos=512012&replicon=Pseudomonas%20fluorescens%20%28strain%20PfO-1%29&default=1) | 509997 | 510012 | - | 1.00 | -7.15 | TCCAGGGCTTTTTCAA | Pfl_0448 | [Pfl_0448 (GE00836564)](http://www.prodoric.de/gene.php?gene_acc=GE00836564) | 9 | intergenic | [SMILE](http://www.prodoric.de/vfp/smile.php?pattern_no=1237392460&gene_acc=GE00836564) |
| [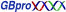](http://www.prodoric.de/gbpro.php?spos=589425&epos=593440&replicon=Pseudomonas%20fluorescens%20%28strain%20PfO-1%29&default=1) | 591425 | 591440 | - | 1.00 | -8.10 | TCAACGCCTTCTTCAC | - | - | - | coding region in gene Pfl_0509 (Pfl_0509) | - |
| [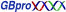](http://www.prodoric.de/gbpro.php?spos=591639&epos=595654&replicon=Pseudomonas%20fluorescens%20%28strain%20PfO-1%29&default=1) | 593639 | 593654 | - | 1.00 | -7.75 | CCAACGGTTTTTTCGC | Pfl_0512 | [Pfl_0512 (GE00836628)](http://www.prodoric.de/gene.php?gene_acc=GE00836628) | 553 | coding region | [SMILE](http://www.prodoric.de/vfp/smile.php?pattern_no=1237392460&gene_acc=GE00836628) |
| [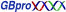](http://www.prodoric.de/gbpro.php?spos=644462&epos=648477&replicon=Pseudomonas%20fluorescens%20%28strain%20PfO-1%29&default=1) | 646462 | 646477 | - | 1.00 | -7.91 | TTCAGGCCTTGTTCAC | - | - | - | coding region in gene Pfl_0552 (Pfl_0552) | - |
| [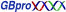](http://www.prodoric.de/gbpro.php?spos=1029242&epos=1033257&replicon=Pseudomonas%20fluorescens%20%28strain%20PfO-1%29&default=1) | 1031242 | 1031257 | + | 1.00 | -6.98 | TTGAAAAAGCCCTGAG | Pfl_0884 | [Pfl_0884 (GE00837009)](http://www.prodoric.de/gene.php?gene_acc=GE00837009) | 162 | coding region | [SMILE](http://www.prodoric.de/vfp/smile.php?pattern_no=1237392460&gene_acc=GE00837009) |
| [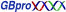](http://www.prodoric.de/gbpro.php?spos=1121918&epos=1125933&replicon=Pseudomonas%20fluorescens%20%28strain%20PfO-1%29&default=1) | 1123918 | 1123933 | - | 1.00 | -7.01 | TTGACGCTTTTTTGAA | Pfl_0963 | [Pfl_0963 (GE00837088)](http://www.prodoric.de/gene.php?gene_acc=GE00837088) | 205 | intergenic | [SMILE](http://www.prodoric.de/vfp/smile.php?pattern_no=1237392460&gene_acc=GE00837088) |
| [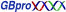](http://www.prodoric.de/gbpro.php?spos=1155181&epos=1159196&replicon=Pseudomonas%20fluorescens%20%28strain%20PfO-1%29&default=1) | 1157181 | 1157196 | - | 1.00 | -7.86 | TTCAGGGCTTTTTCGC | - | - | - | coding region in gene Pfl_0995 (Pfl_0995) | - |
| [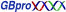](http://www.prodoric.de/gbpro.php?spos=1322275&epos=1326290&replicon=Pseudomonas%20fluorescens%20%28strain%20PfO-1%29&default=1) | 1324275 | 1324290 | - | 1.00 | -7.64 | TCTACTCCTTTTTCAC | Pfl_1148 | [Pfl_1148 (GE00837273)](http://www.prodoric.de/gene.php?gene_acc=GE00837273) | 6 | intergenic | [SMILE](http://www.prodoric.de/vfp/smile.php?pattern_no=1237392460&gene_acc=GE00837273) |
| [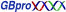](http://www.prodoric.de/gbpro.php?spos=1539803&epos=1543818&replicon=Pseudomonas%20fluorescens%20%28strain%20PfO-1%29&default=1) | 1541803 | 1541818 | - | 1.00 | -6.82 | TTCAGGCTTTTTTCAG | Pfl_1366 | [Pfl_1366 (GE00837494)](http://www.prodoric.de/gene.php?gene_acc=GE00837494) | 323 | coding region | [SMILE](http://www.prodoric.de/vfp/smile.php?pattern_no=1237392460&gene_acc=GE00837494) |
| [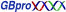](http://www.prodoric.de/gbpro.php?spos=1539803&epos=1543818&replicon=Pseudomonas%20fluorescens%20%28strain%20PfO-1%29&default=1) | 1541803 | 1541818 | - | 1.00 | -6.82 | TTCAGGCTTTTTTCAG | Pfl_1368 | [Pfl_1368 (GE00837496)](http://www.prodoric.de/gene.php?gene_acc=GE00837496) | 32 | coding region | [SMILE](http://www.prodoric.de/vfp/smile.php?pattern_no=1237392460&gene_acc=GE00837496) |
| [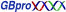](http://www.prodoric.de/gbpro.php?spos=1698440&epos=1702455&replicon=Pseudomonas%20fluorescens%20%28strain%20PfO-1%29&default=1) | 1700440 | 1700455 | - | 1.00 | -7.76 | TCCAGGCCTTTTTCCC | - | - | - | coding region in gene Pfl_1513 (Pfl_1513) | - |
| [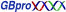](http://www.prodoric.de/gbpro.php?spos=1722009&epos=1726024&replicon=Pseudomonas%20fluorescens%20%28strain%20PfO-1%29&default=1) | 1724009 | 1724024 | + | 1.00 | -7.08 | GTCAAAAAATTGCGGG | Pfl_1535 | [Pfl_1535 (GE00837664)](http://www.prodoric.de/gene.php?gene_acc=GE00837664) | 20 | intergenic | [SMILE](http://www.prodoric.de/vfp/smile.php?pattern_no=1237392460&gene_acc=GE00837664) |
| [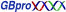](http://www.prodoric.de/gbpro.php?spos=1726641&epos=1730656&replicon=Pseudomonas%20fluorescens%20%28strain%20PfO-1%29&default=1) | 1728641 | 1728656 | - | 1.00 | -7.91 | TTGAGGCCTTCTTCAC | - | - | - | coding region in gene Pfl_1539 (Pfl_1539) | - |
| [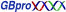](http://www.prodoric.de/gbpro.php?spos=1844909&epos=1848924&replicon=Pseudomonas%20fluorescens%20%28strain%20PfO-1%29&default=1) | 1846909 | 1846924 | - | 0.00 | -6.80 | CTGACGATTTTTTCAC | Pfl_1658 | [Pfl_1658 (GE00837787)](http://www.prodoric.de/gene.php?gene_acc=GE00837787) | 190 | intergenic | [SMILE](http://www.prodoric.de/vfp/smile.php?pattern_no=1237392460&gene_acc=GE00837787) |
| [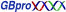](http://www.prodoric.de/gbpro.php?spos=2224873&epos=2228888&replicon=Pseudomonas%20fluorescens%20%28strain%20PfO-1%29&default=1) | 2226873 | 2226888 | - | 1.00 | -7.57 | CTGAAACCTTTTTCAC | - | - | - | coding region in gene Pfl_1952 (Pfl_1952) | - |
| [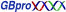](http://www.prodoric.de/gbpro.php?spos=2342743&epos=2346758&replicon=Pseudomonas%20fluorescens%20%28strain%20PfO-1%29&default=1) | 2344743 | 2344758 | - | 1.00 | -7.58 | TCGAGACTTTTTTCGC | Pfl_2056 | [Pfl_2056 (GE00838200)](http://www.prodoric.de/gene.php?gene_acc=GE00838200) | 96 | coding region | [SMILE](http://www.prodoric.de/vfp/smile.php?pattern_no=1237392460&gene_acc=GE00838200) |
| [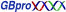](http://www.prodoric.de/gbpro.php?spos=2381763&epos=2385778&replicon=Pseudomonas%20fluorescens%20%28strain%20PfO-1%29&default=1) | 2383763 | 2383778 | - | 1.00 | -7.80 | CTGCCGGTTTTTTCAC | - | - | - | coding region in gene Pfl_2097 (Pfl_2097) | - |
| [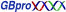](http://www.prodoric.de/gbpro.php?spos=2488642&epos=2492657&replicon=Pseudomonas%20fluorescens%20%28strain%20PfO-1%29&default=1) | 2490642 | 2490657 | - | 1.00 | -7.36 | CCCACACTTTTATGAC | Pfl_2203 | [Pfl_2203 (GE00838347)](http://www.prodoric.de/gene.php?gene_acc=GE00838347) | 27 | intergenic | [SMILE](http://www.prodoric.de/vfp/smile.php?pattern_no=1237392460&gene_acc=GE00838347) |
| [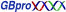](http://www.prodoric.de/gbpro.php?spos=2743151&epos=2747166&replicon=Pseudomonas%20fluorescens%20%28strain%20PfO-1%29&default=1) | 2745151 | 2745166 | + | 1.00 | -7.67 | GTCATAAAAACCTCAA | Pfl_2385 | [Pfl_2385 (GE00838535)](http://www.prodoric.de/gene.php?gene_acc=GE00838535) | 344 | coding region | [SMILE](http://www.prodoric.de/vfp/smile.php?pattern_no=1237392460&gene_acc=GE00838535) |
| [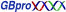](http://www.prodoric.de/gbpro.php?spos=2808977&epos=2812992&replicon=Pseudomonas%20fluorescens%20%28strain%20PfO-1%29&default=1) | 2810977 | 2810992 | - | 1.00 | -7.97 | TCGACCCCTTTTTCAC | Pfl_2457 | [Pfl_2457 (GE00838606)](http://www.prodoric.de/gene.php?gene_acc=GE00838606) | 199 | coding region | [SMILE](http://www.prodoric.de/vfp/smile.php?pattern_no=1237392460&gene_acc=GE00838606) |
| [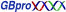](http://www.prodoric.de/gbpro.php?spos=2816192&epos=2820207&replicon=Pseudomonas%20fluorescens%20%28strain%20PfO-1%29&default=1) | 2818192 | 2818207 | - | 1.00 | -6.96 | TTCACATTTCTTTCAC | Pfl_2462 | [Pfl_2462 (GE00838611)](http://www.prodoric.de/gene.php?gene_acc=GE00838611) | 94 | intergenic | [SMILE](http://www.prodoric.de/vfp/smile.php?pattern_no=1237392460&gene_acc=GE00838611) |
| [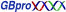](http://www.prodoric.de/gbpro.php?spos=2816192&epos=2820207&replicon=Pseudomonas%20fluorescens%20%28strain%20PfO-1%29&default=1) | 2818192 | 2818207 | - | 1.00 | -6.96 | TTCACATTTCTTTCAC | Pfl_2463 | [Pfl_2463 (GE00838612)](http://www.prodoric.de/gene.php?gene_acc=GE00838612) | 148 | intergenic | [SMILE](http://www.prodoric.de/vfp/smile.php?pattern_no=1237392460&gene_acc=GE00838612) |
| [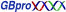](http://www.prodoric.de/gbpro.php?spos=2966059&epos=2970074&replicon=Pseudomonas%20fluorescens%20%28strain%20PfO-1%29&default=1) | 2968059 | 2968074 | - | 1.00 | -7.67 | TTGAGTTCATTTTCAC | - | - | - | coding region in gene Pfl_2589 (Pfl_2589) | - |
| [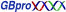](http://www.prodoric.de/gbpro.php?spos=3106075&epos=3110090&replicon=Pseudomonas%20fluorescens%20%28strain%20PfO-1%29&default=1) | 3108075 | 3108090 | - | 1.00 | -7.69 | CCCAGATCTTTTTCAT | - | - | - | coding region in gene Pfl_2698 (Pfl_2698) | - |
| [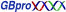](http://www.prodoric.de/gbpro.php?spos=3582880&epos=3586895&replicon=Pseudomonas%20fluorescens%20%28strain%20PfO-1%29&default=1) | 3584880 | 3584895 | - | 1.00 | -7.47 | CCGACGACTTTTTCAG | Pfl_3123 | [Pfl_3123 (GE00839274)](http://www.prodoric.de/gene.php?gene_acc=GE00839274) | 173 | intergenic | [SMILE](http://www.prodoric.de/vfp/smile.php?pattern_no=1237392460&gene_acc=GE00839274) |
|  | 3889777 | 3889792 | + | 1.00 | -8.26 | GTGAAAACAGCGTCGA | - | - | - | coding region in gene Pfl_3410 (Pfl_3410) | - |
|  | 3928171 | 3928186 | - | 1.00 | -7.74 | GCCAGTCCTTTTTCAC | - | - | - | coding region in gene Pfl_3443 (Pfl_3443) | - |
|  | 3980847 | 3980862 | - | 1.00 | -7.12 | TCAACACTTTTTACAC | Pfl_3500 | [Pfl_3500 (GE00839651)](http://www.prodoric.de/gene.php?gene_acc=GE00839651) | 254 | intergenic | [SMILE](http://www.prodoric.de/vfp/smile.php?pattern_no=1237392460&gene_acc=GE00839651) |
|  | 4031156 | 4031171 | - | 1.00 | -7.88 | TCAACTGGTTTTTCAC | - | - | - | coding region in gene Pfl_3559 (Pfl_3559) | - |
|  | 4049218 | 4049233 | - | 1.00 | -7.49 | CTGACGACTTTTTCAT | Pfl_3582 | [Pfl_3582 (GE00839733)](http://www.prodoric.de/gene.php?gene_acc=GE00839733) | 144 | coding region | [SMILE](http://www.prodoric.de/vfp/smile.php?pattern_no=1237392460&gene_acc=GE00839733) |
|  | 4216661 | 4216676 | - | 1.00 | -7.55 | TCCAGACCTTTTTGTC | - | - | - | coding region in gene Pfl_3731 (Pfl_3731) | - |
|  | 4337625 | 4337640 | + | 1.00 | -8.09 | GTCAACAAGCCGTTGA | Pfl_3834 | [Pfl_3834 (GE00839990)](http://www.prodoric.de/gene.php?gene_acc=GE00839990) | 458 | coding region | [SMILE](http://www.prodoric.de/vfp/smile.php?pattern_no=1237392460&gene_acc=GE00839990) |
|  | 4577703 | 4577718 | - | 1.00 | -6.72 | TTGACGGGTTTTTCAC | - | - | - | coding region in gene Pfl_4052 (Pfl_4052) | - |
|  | 4596798 | 4596813 | - | 1.00 | -7.43 | CCGAGTGTTTTTTGAA | Pfl_4070 | [Pfl_4070 (GE00840230)](http://www.prodoric.de/gene.php?gene_acc=GE00840230) | 286 | coding region | [SMILE](http://www.prodoric.de/vfp/smile.php?pattern_no=1237392460&gene_acc=GE00840230) |
|  | 5071800 | 5071815 | - | 1.00 | -6.74 | ATGACGTTTTTTTCAC | Pfl_4496 | [Pfl_4496 (GE00840668)](http://www.prodoric.de/gene.php?gene_acc=GE00840668) | 70 | intergenic | [SMILE](http://www.prodoric.de/vfp/smile.php?pattern_no=1237392460&gene_acc=GE00840668) |
|  | 5261351 | 5261366 | - | 1.00 | -7.45 | CCAGGTGTTTTTTCAC | - | - | - | coding region in gene Pfl_4662 (Pfl_4662) | - |
|  | 5320007 | 5320022 | - | 1.00 | -7.96 | CTGACGGCTTTTTGAT | Pfl_4710 | [Pfl_4710 (GE00840883)](http://www.prodoric.de/gene.php?gene_acc=GE00840883) | 465 | coding region | [SMILE](http://www.prodoric.de/vfp/smile.php?pattern_no=1237392460&gene_acc=GE00840883) |
|  | 5416484 | 5416499 | + | 1.00 | -7.77 | GGGAAAAAACTGTTGG | Pfl_4800 | [Pfl_4800 (GE00840980)](http://www.prodoric.de/gene.php?gene_acc=GE00840980) | 226 | coding region | [SMILE](http://www.prodoric.de/vfp/smile.php?pattern_no=1237392460&gene_acc=GE00840980) |
|  | 5425067 | 5425082 | - | 1.00 | -7.29 | GTAACAGTTTTTTCAC | - | - | - | non-coding region between gene Pfl_4808 (Pfl_4808) and Pfl_4809 (Pfl_4809) | - |
|  | 5443517 | 5443532 | - | 1.00 | -7.19 | TCCAGGGTTTTTCCAC | Pfl_4824 | [Pfl_4824 (GE00841004)](http://www.prodoric.de/gene.php?gene_acc=GE00841004) | 593 | coding region | [SMILE](http://www.prodoric.de/vfp/smile.php?pattern_no=1237392460&gene_acc=GE00841004) |
|  | 5481606 | 5481621 | - | 1.00 | -7.69 | CTCAGACTTTTCTGAC | Pfl_4863 | [Pfl_4863 (GE00841047)](http://www.prodoric.de/gene.php?gene_acc=GE00841047) | 207 | coding region | [SMILE](http://www.prodoric.de/vfp/smile.php?pattern_no=1237392460&gene_acc=GE00841047) |
|  | 5645897 | 5645912 | - | 1.00 | -7.66 | TCGAAGGTTTTTTCAC | - | - | - | coding region in gene Pfl_5008 (Pfl_5008) | - |
|  | 5680129 | 5680144 | - | 1.00 | -7.81 | TCGACGGTTTTTTCGC | - | - | - | coding region in gene Pfl_5043 (Pfl_5043) | - |
|  | 5806288 | 5806303 | + | 1.00 | -7.14 | TTGAAAAAAACCTCGA | Pfl_5164 | [Pfl_5164 (GE00841358)](http://www.prodoric.de/gene.php?gene_acc=GE00841358) | 0 | intergenic | [SMILE](http://www.prodoric.de/vfp/smile.php?pattern_no=1237392460&gene_acc=GE00841358) |
|  | 5806288 | 5806303 | + | 1.00 | -7.14 | TTGAAAAAAACCTCGA | Pfl_5165 | [Pfl_5165 (GE00841359)](http://www.prodoric.de/gene.php?gene_acc=GE00841359) | 155 | intergenic | [SMILE](http://www.prodoric.de/vfp/smile.php?pattern_no=1237392460&gene_acc=GE00841359) |
|  | 5942260 | 5942275 | - | 1.00 | -7.66 | CTGACACTGTTTTGAC | Pfl_5281 | [Pfl_5281 (GE00841475)](http://www.prodoric.de/gene.php?gene_acc=GE00841475) | 87 | intergenic | [SMILE](http://www.prodoric.de/vfp/smile.php?pattern_no=1237392460&gene_acc=GE00841475) |
|  | 6045818 | 6045833 | - | 1.00 | -7.33 | CCCACAGTTTTTTCAT | - | - | - | non-coding region between gene Pfl_5382 (Pfl_5382) and Pfl_5383 (Pfl_5383) | - |
|  | 6047923 | 6047938 | - | 1.00 | -7.36 | TTGCCGTTTTTTTCAC | Pfl_5384 | [Pfl_5384 (GE00841578)](http://www.prodoric.de/gene.php?gene_acc=GE00841578) | 92 | intergenic | [SMILE](http://www.prodoric.de/vfp/smile.php?pattern_no=1237392460&gene_acc=GE00841578) |
|  | 6112865 | 6112880 | - | 1.00 | -7.18 | TCAAGAAGTTTTTGAC | - | - | - | coding region in gene Pfl_5446 (Pfl_5446) | - |
|  | 6217000 | 6217015 | - | 1.00 | -8.21 | TCGAGGACTTTCTGAC | Pfl_5549 | [Pfl_5549 (GE00841743)](http://www.prodoric.de/gene.php?gene_acc=GE00841743) | 317 | coding region | [SMILE](http://www.prodoric.de/vfp/smile.php?pattern_no=1237392460&gene_acc=GE00841743) |
|  | 6272653 | 6272668 | - | 1.00 | -7.33 | CCGTGTGTTTTTTGAC | - | - | - | coding region in gene Pfl_5598 (Pfl_5598) | - |

Pfl_1183 is orthologuous to *P. putida* PP1636, diacylglycerol kinase

Pfl_1658 is orthologuous to *P. putida* PP1692, hypothetical protein

Pfl_4496 is orthologuous to *P. putida* PP0903, conserved hypothetical protein

Pfl_4498 is orthologuous to *P. putida* PP0901, DNA-binding response regulator ColR

Pfl_4500 is orthologuous to *P. putida* PP0900, PAP2 family protein

Pfl_4564 is orthologuous to *P. putida* PP1058, conserved hypothetical protein

Pfl_4744 is orthologuous to *P. putida* PP0737, conserved hypothetical protein

Pfl_5384 is orthologuous to *P. putida* PP5152, conserved hypothetical protein
