# Supplementary material for: Identification of ColR binding consensus and prediction of regulon of ColRS two-component system
Source: BMC Mol Biol. 2009 May 16;10:46. doi: 10.1186/1471-2199-10-46 (PMC2689224; doi:10.1186/1471-2199-10-46)
Supplement: Additional file 3 — Oligonucleotides. Sequences of oligonucleotides used in a current study. [file 1471-2199-10-46-S3.doc]

### Additional file 3

### Supplementary table 4. Sequences of oligonucleotides used in a current study.

| **Purpose** | **Oligonucleotide designation** | **Oligonucleotide sequence** | **PCR product cleaved with** | **p9TT_B_lacZ cleaved with** |
| --- | --- | --- | --- | --- |
| Cloning of promoters into p9TT_B_lacZ | 0035alg  0036ees | TGGGATCCCGTTGCTATGAAGCCG  TGGGATCCACCGGGTCATGCATGC | BamHI | BamHI |
|  | 0736lopp  0737start | GCGGATCCTCGGCTGCACCCAT  CAGGATCCGCTGCCAAGCCGAGCA | BamHI | BamHI |
|  | colREcoRV  900Kpn | CCAGGATATCGCGGTTGTCTT  CAGGGGTACCGCGGGGACA | EcoRV | SmaI |
|  | orf222prom  orf222alg | AAGCTCGAGCCTGGAAGCCAACGGT  TTCAAGCTTGATGGGGCTACG | XhoI/HindIII | XhoI/HindIII |
|  | 1635lopp  1636alg | CGAAAGCTTGCTGAACAAGCAGATC  CTGAAGCTTGAATGGCGATGTCATGT | HindIII | HindIII |
|  | 2560start  2561start | TCGGATCCGGTGCGTGGTGGCTTAGG  ATGGATCCGGAGATGACACAGGGC | BamHI | BamHI |
|  | 3765stop  gloAstart | GAAAAGCTTGGGGCCGCCACGGTAGA  CAAAGCTTGCAGATCGTGCAGGCTC | HindIII | HindIII |
| Verification of promoter orientation in p9TT_B_lacZ | lacZseq | GGGGATGTGCTGCAAGGCG |  |  |
| Generation of DNA probes for DNase I footprint | 0035alg  0036ees | TGGGATCCCGTTGCTATGAAGCCG  TGGGATCCACCGGGTCATGCATGC |  |  |
|  | 0737Kpn  0737start | TTGGGTACCAATGATCTGTTGGCCGAA  CAGGATCCGCTGCCAAGCCGAGCA |  |  |
|  | colREcoRV  colRfootpr | CCAGGATATCGCGGTTGTCTT  AGGTACAGCCAGTAGTTGATC |  |  |
|  | orf222start  orf222alg | ACCCTGACATCCACCTTGC  TTCAAGCTTGATGGGGCTACG |  |  |
|  | 1635lopp  1636alg | CGAAAGCTTGCTGAACAAGCAGATC  CTGAAGCTTGAATGGCGATGTCATGT |  |  |
|  | 2560Fw  2560Rev | GTGGATCCCACAGACAGCAGCGTA  AGGGATCCTGTAAACCGTGCGCAACA |  |  |
|  | 3765stop  gloAfootpr | GAAAAGCTTGGGGCCGCCACGGTAGA  GCTGGCCATGATACGGCAG |  |  |
|  | 0267start  0268gatc | GGGTACAAGCATGGGCACAT  ATCCAGCCGGGTGGGCCGA |  |  |
